# Uniaxial spin texture in a superconducting electron gas revealed by exchange interactions

**DOI:** 10.1126/sciadv.aeb1601

**Published:** 2026-05-08

**Authors:** Junyi Yang, Changjiang Liu, Xianjing Zhou, Hanyu Hou, Kaijun Yin, Jianguo Wen, John Pearson, Alexey Suslov, Dafei Jin, Jidong S. Jiang, Ulrich Welp, Jian-Min Zuo, Michael R. Norman, Anand Bhattacharya

**Affiliations:** ^1^ Materials Science Division, Argonne National Laboratory, Lemont, IL, USA.; ^2^ Department of Physics, University at Buffalo, SUNY, Buffalo, NY, USA.; ^3^ Center for Nanoscale Materials, Argonne National Laboratory, Lemont, IL, USA.; ^4^ National High Field Magnetic Laboratory, Tallahassee, FL, USA.; ^5^ Florida State University, Tallahassee, FL, USA.; ^6^ Department of Material Science and Engineering, University of Illinois at Urbana-Champaign, Urbana, IL, USA.; ^7^ University of Notre Dame, Notre Dame, IN, USA.

## Abstract

Two-dimensional (2D) superconductors with spin-textured Fermi surfaces can be a platform for realizing unconventional pairing states and are of substantial interest in the context of quantum information science and superconducting spintronics/orbitronics. We observed an unusual in-plane uniaxial anisotropy in the superconducting 2D electron gas (2DEG) formed at EuO*_x_*/KTaO_3_ (110) interfaces. This anisotropy is not evident in AlO*_x_*/KTaO_3_ (110) where the overlayer is nonmagnetic. Our results are consistent with a highly anisotropic “half-Rashba” spin-textured Fermi surface in 2DEGs formed at the KTaO_3_ (110) interface that is hidden from external magnetic fields due to a near cancellation between orbital and spin moments but revealed by exchange interactions of the electrons in the 2DEG with Eu moments near the EuO*_x_*/KTaO_3_ (110) interface. The interactions between the uniaxial spin texture and the magnetic overlayer offer previously unexplored ways to explore the interplay between magnetism and 2D superconductivity.

## Teaser

Exchange interaction with rare-earth ions reveals a hidden in-plane uniaxial spin texture in superconducting KTaO_3_ 2DEGs.

## INTRODUCTION

The interplay of large spin-orbit coupling (SOC) with crystalline symmetry breaking can result in nontrivial spin textures for electrons at the Fermi surface of a two-dimensional electron gas (2DEG) (*1*, *2*). Broken inversion symmetry along the out-of-plane direction in 2DEGs leads to a “Rashba” spin texture, where spins are locked in-plane with opposite helicity on spin-split Fermi surfaces (*3*), and such asymmetric SOC also allows for superconductivity with mixed parity (*4*, *5*). The presence of strong SOC with in-plane inversion symmetry breaking or broken orbital degeneracy can lead to an “Ising” spin texture that locks spins along the out-of-plane direction, and, for superconducting 2DEGs, this results in large in-plane critical fields (*6*–*8*) well in excess of the Pauli limit and may lead to unconventional superconductivity (*9*). These spin textures are nominally not expected to give rise to an anisotropic response to in-plane magnetic fields. Rather, such a response is often associated with effective mass anisotropy, nematicity (*10*), and superconducting order parameters with mixed-parity (*11*). On the other hand, an asymmetric strong SOC in a 2DEG with reduced symmetry can result in an anisotropic Rashba interaction, where the spins may have an in-plane anisotropy or even be locked-in along an axis for a “half-Rashba” interaction (*12*). Such a uniaxial spin texture of the Fermi surface would lead to superconductivity with inherent in-plane anisotropy, which is of both fundamental interest (*13*) and may also be relevant for applications in quantum information science (*14*), superconducting spintronics, and orbitronics (*15*, *16*).

**Fig. 1.**
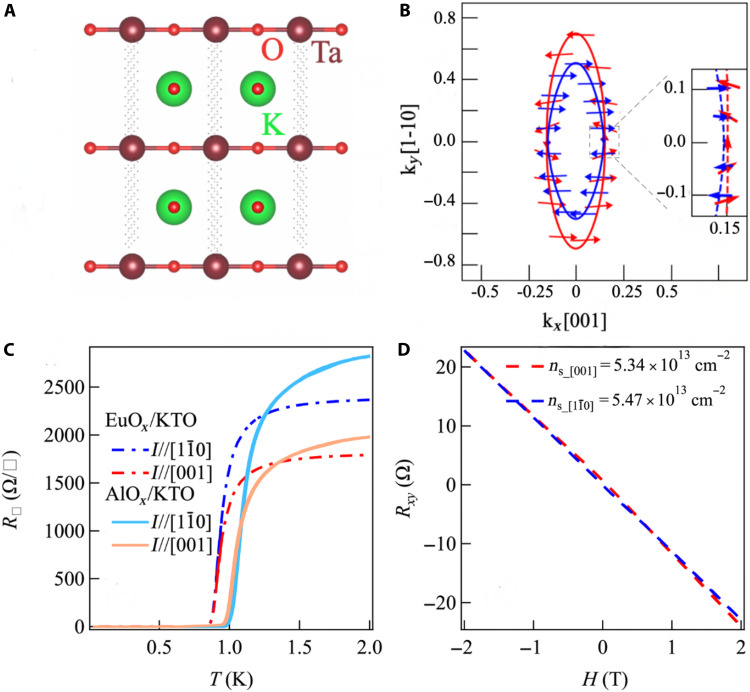
Uniaxial spin texture for KTO (110) interface. (**A**) Schematic diagram of KTO (110). (**B**) KTO (110) Fermi surface derived from d*_xz_*/d*_yz_* states, with its anisotropic spin texture. The length of the arrows is related to the spin vector size, with the inset showing an expanded view around [001]. The two Rashba spin-split surfaces are shown in red and blue, assuming an exaggerated spin-splitting *t*_R_ = 20 meV to better illustrate the spin textures on the inner and outer Fermi surfaces. We assume *t*_R_ = 2 meV in all other calculations. (**C**) Temperature dependence of the sheet resistance of EuO*_x_*/KTO and AlO*_x_*/KTO with current applied along two different in-plane directions. The light blue solid line denotes the current along [1$\bar{1}$0], and the light red solid line denotes the current along [001] for AlO*_x_*/KTO. The blue dashed line denotes the current along [1$\bar{1}$0], and the red dashed line denotes the current along [001] for EuO*_x_*/KTO. (**D**) Hall measurements for Hall bar devices along the [001]/[1$\bar{1}$0] directions, respectively. The carrier density along both orientations is very close.

Superconductivity was recently discovered in 2DEGs formed at interfaces of KTaO_3_ (KTO) (*17*). Notably, the critical temperature (*T*_c_) in KTO 2DEGs was found to strongly depend on the orientation of the interface (*17*–*19*), and it was proposed that the orientation dependence of *T*_c_ is due to varying levels of degeneracy in the *t*_2g_ manifold of the Ta-5d bands in quantum-confined 2DEGs formed on different crystalline facets of KTO (*19*). For the KTO (110) interface (Fig. 1A), the degenerate d*_xz_*/d*_yz_* states at the Γ point are lower in energy than d*_xy_* due to confinement along the [110] axis, and they form an anisotropic Fermi surface with larger/smaller effective mass along the [1$\bar{1}$0]/[001] axis (*20*), respectively. Furthermore, the d*_xz_*/d*_yz_* states at Γ form the combination d*_yz_* ± id*_xz_* with an orbital angular momentum axis oriented along the in-plane [001] direction. Due to SOC, the spins antialign to these orbital moments, and this gives rise to an in-plane anisotropy for both orbital and spin textures along [001] with no spin canting along the out-of-plane direction. Additionally, broken inversion symmetry at the KTO (110) interface gives rise to Rashba splitting of bands formed from these states, which, along with the uniaxial anisotropy, induces a dominant $k_{y}{\hat{\sigma }}_{x}$ half-Rashba texture due to spin-momentum locking (here, *x* refers to [001] and *y* to [1$\bar{1}$0]) (Fig. 1B). We note that this uniaxial anisotropy is unique to the KTO (110) interface (*21*, *22*) and distinct from the more isotropic spin textures calculated for KTO (111) (*23*) and KTO (001) (*24*) interfacial 2DEGs. Last, as is well-known, for single-electron occupancy in the *t*_2g_ manifold, the orbital and spin moments are “antialigned,” which leads to a strongly reduced *g*-factor (*25*), and a reduced Zeeman coupling of external magnetic fields (*26*) to the electronic spin texture. Thus, KTO interfacial 2DEGs are nominally expected to have a relatively weak response to in-plane magnetic fields.

## RESULTS

In this work, we report on a uniaxial in-plane spin texture for KTO_3_ (110) interfaces, which is revealed by the anisotropy of the in-plane upper critical field *H*_c_ as well as the normal state magnetoresistance for EuO*_x_*/KTO (110) 2DEGs, where the EuO*_x_* overlayer is magnetic. We fabricated Hall bar devices on both AlO*_x_*/KTO (110) and EuO*_x_*/KTO (110) (fig. S1) 2DEGs. Here, AlO*_x_* is nonmagnetic, while EuO*_x_* is ferromagnetic with *T*_Curie_ of ~70 K. Figure 1C shows the temperature dependence of the sheet resistance ($R_{□}$) for EuO*_x_*/KTO with current along [001]/$\left[1\bar{1}0\right]$, respectively. The crystallographic axis for each direction is determined through x-ray diffraction (fig. S2). Superconductivity is observed for both samples below 800 mK. The normal state $R_{□}$ for a current along [1$\bar{1}$0] is found to be larger than that for a current along [001] by a factor of 1.5 for all samples. These observations are consistent with a highly anisotropic Fermi surface of KTO (110) (Fig. 1B) (*20*), where the effective mass along the [1$\bar{1}$0] direction is larger than that along [001]. Hall measurements (Fig. 1D) along these two crystallographic directions are nearly identical, and, presumably, the anisotropic $R_{□}$ is due to the anisotropy in electron mobility. We note that, for LAO/STO (110), a similar anisotropy in $R_{□}$ is observed, although it changes as a function of carrier density (*27*–*29*) due to occupation of the d*_xy_* bands at higher densities. In contrast, for our KTO (110) 2DEGs, the anisotropy of the normal state $R_{□}$ is found to be similar for both low and high densities. This may imply that the d*_xy_* bands are not occupied or have fewer carriers than the d*_xz_*/d*_yz_* band in our KTO (110) 2DEGs, presumably due to the stronger confinement effects in KTO (110) relative to STO (110). However, we note that recent angle-resolved photoemission experiments on doped KTO (110) surfaces with higher carrier density (~7 × 10^13^/cm^2^) do observe the occupation of the higher lying d*_xy_* bands (*20*).

### Anisotropic superconductor-ferromagnet interaction in EuO*_x_*/KTO (110) near *T*_c_

**Fig. 2.**
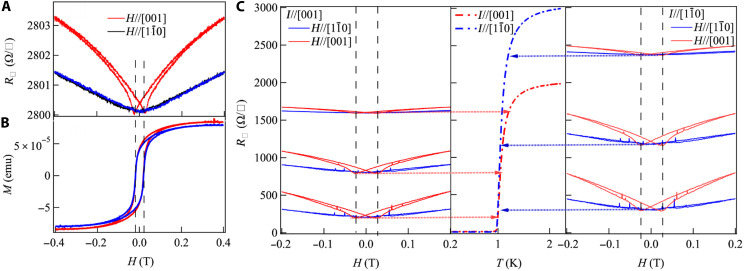
Anisotropic magnetoresistance in EuO/KTO (110). (**A**) Field dependence of the sheet resistance for EuO/KTO (110) at 2 K. The red solid line denotes a field along [001], and the blue solid line denotes a field along [1$\bar{1}$0]. (**B**) Field dependence of the magnetic moment for EuO/KTO (110) at 2 K, showing a nearly isotropic coercivity. The red solid line denotes a field along [001] and the blue solid line denotes a field along [1$\bar{1}$0]. (**C**) Field dependence of the sheet resistance for EuO/KTO (110) with current applied along different crystal axes. The left panel shows the field dependence of the sheet resistance when the current is applied along [001]. The right panel shows the field dependence of the sheet resistance when the current is applied along [1$\bar{1}$0]. The red solid line denotes a field along [001], and the blue solid line denotes a field along [1$\bar{1}$0]. The central panel shows the temperature dependence of the sheet resistance for *I*//[001] (red solid dashed line) and *I*//[1$\bar{1}$0] (blue solid dashed line). The arrows are used to indicate the different temperatures that the field dependence of the resistance is measured*.*

To explore the anisotropic spin texture in KTO (110), we first measured the in-plane magnetic field dependence of $R_{□}$ for EuO*_x_*/KTO (110). We find evidence for coupling between conduction electrons in the 2DEGs and the magnetic EuO*_x_* overlayer in measurements of $R_{□}$(H) at 2 K (above *T*_c_), where we observe hysteretic behavior at low fields for *H*//[001] (Fig. 2A). Notably, the magnetoresistance (MR) = $\frac{R\left(H\right)-R\left(0\right)}{R\left(0\right)}$ and the apparent hysteresis for *H*//[001] is much larger than that for *H*//[1$\bar{1}$0]. The hysteretic minima in $R_{□}$(*H*) at ±115 Oe are consistent with the in-plane coercive field of the EuO*_x_* overlayer of ~115 Oe from magnetization measurements on this sample. The magnetization-versus-*H* measurements of the EuO*_x_* overlayer are nearly identical for *H*//[001] and *H*//[1$\bar{1}$0], implying that the observed MR anisotropy is inherent to the KTO 2DEG and not the magnetization of the EuO*_x_* overlayer. As the temperature is lowered toward *T*_c_, the MR rapidly increases in magnitude, approaching ~130% for *H*//[001], and ~47% for *H*//[1$\bar{1}$0] for *H* = 0.2 T at a temperature of ~1 K. We also find that the highly anisotropic MR is independent of the current direction, from our measurements on Hall bars oriented along [001] and [1$\bar{1}$0] on the same sample, indicating that the anisotropy in the MR only depends on the orientation of the magnetic field with respect to the crystal lattice. We also note a weak anomaly in the temperature dependence of the MR near the Curie temperature of the EuO*_x_* overlayer (fig. S3), further evidence for its coupling to electrons in the KTO 2DEG.

We note that a hysteretic MR has been observed in 2DEGs formed at LaAlO_3_/SrTiO_3_, GdTiO_3_/SrTiO_3_, and LaTiO_3_/SrTiO_3_ interfaces when magnetism is induced (*30*–*32*), and recent work also shows hysteretic MR at KTO interfaces (*33*, *34*). However, the in-plane anisotropy in MR that we observe has not been reported earlier to the best of our knowledge. The anisotropy of the MR near the superconducting transition in Fig. 2 is consistent with a pair-breaking effect that arises from interfacial exchange coupling of ferromagnetic Eu spins in EuO*_x_* with the highly anisotropic in-plane uniaxial spin texture of KTO (110) 2DEGs. However, this low-field MR for in-plane fields, including the hysteretic behavior, is no longer observed once we cool below *T*_c_ as the experimentally measured in-plane *H*_c_ increases far above the coercivity field of the EuO*_x_* overlayer. This also implies that the interfacial exchange coupling between the EuO*_x_* and the KTO (110) 2DEG is relatively weak, being effective only in the transition region near *T*_c_ where the conductivity is enhanced by superconducting fluctuations, but not below *T*_c_ where the superconductivity is more robust.

### Anisotropic in-plane upper critical field

**Fig. 3.**
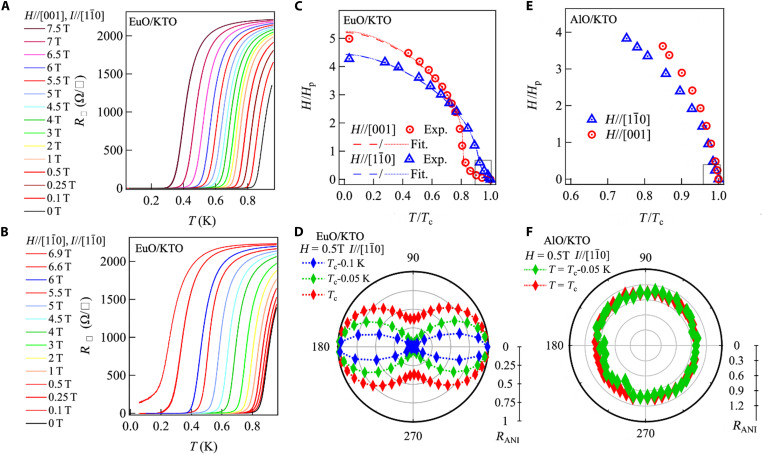
Anisotropic critical field for KTO (110). Temperature dependence of the sheet resistance of EuO*_x_*/KTO for different in-plane fields when (**A**) the field is applied along [001] and (**B**) when the field is applied along [1$\bar{1}$0]. (**C**) Temperature dependence of the critical field of EuO*_x_*/KTO for the [1$\bar{1}$0] and [001] field directions. *T*_c_ is normalized to its zero-field value, and *H*_c_ is normalized to the Pauli paramagnetic field. The solid/dashed line denotes fits based on the KLB/WHH models described in the text. (**D**) The angular dependence of the normalized sheet resistance of EuO*_x_*/KTO for an in-plane field of 0.5 T for three different temperatures. A lower *R*_ANI_ would imply a higher value of H_c_. (**E**) Temperature dependence of the critical field of AlO*_x_*/KTO for the [1$\bar{1}$0] and [001] field directions. (**F**) The angular dependence with respect to the field direction of the normalized sheet resistance for AlO*_x_*/KTO with a field of 0.5 T at two different temperatures. A higher *R*_ANI_ would imply a lower value of *H*_c_.

We also observe a striking anisotropy in the in-plane *H*_c_ of EuO*_x_*/KTO (110) 2DEGs as we cool below *T*_c_. Figure 3 (A and B) shows the temperature dependence of $R_{□}$ when the field is applied along the [001] and [1$\bar{1}$0] directions. Figure 3C summarizes the *T* dependence of *H*_c_ for both directions. *T*_c_ is taken as the inflection point in the temperature dependence of *R*_□_, normalized to its zero-field value. *H*_c_ is normalized to the conventional Pauli paramagnetic field $\mu_{0}H_{P}\left[T\right]=1.86\;T_{c}\;\left[K\right]$ (*35*). Note that the actual paramagnetic limiting field can be substantially higher both due to spin-orbit scattering and a reduced g-factor (*36*, *37*). At the lowest temperatures, in EuO*_x_*/KTO (110), we find that *H*_c_//[1$\bar{1}$0] < *H*_c_//[001] where $\frac{H_{c}//\left[1\bar{1}0\right]}{H_{c}//\left[001\right]}\sim 0.8$ (Fig. 3 and fig. S9D). However, closer to *T*_c_, this ratio strongly inverts and $\frac{H_{c}//\left[1\bar{1}0\right]}{H_{c}//\left[001\right]}>7$ at *T* = 0.9 *T*_c_. This switching of anisotropy in *H*_c_ is observed in several EuO*_x_*/KTO (110) samples, for a range of *T*_c_ values (figs. S5 to S7). The twofold nature of the anisotropy in *H*_c_ close to *T*_c_ is evident in the angular dependence of the normalized $R_{□}$, *R*_ANI_(φ) = $R_{□}$(φ)*/*$R_{□}$(φ *=* 0) (here, φ *= 0* for *H*// [001]), in a 0.5 T in-plane magnetic field (Fig. 3D). A dumbbell-shaped *R*_ANI_(φ) is observed with minima for magnetic fields along [1$\bar{1}$0], implying *H*_c_//[1$\bar{1}$0] > *H*_c_//[001].

In addition, we also measured the dependence of *T*_c_ on in-plane magnetic fields along [001] and [1$\bar{1}$0] for AlO*_x_*/KTO (110) samples (fig. S8). Figure 3E summarizes the *T* dependence of *H*_c_ for the [001] and [1$\bar{1}$0] directions for AlO*_x_*/KTO (110) with *T*_c_ = 1080 mK. In contrast to EuO*_x_*/KTO (110), for AlO*_x_*/KTO(110), the ratio of upper critical fields $\frac{H_{c}//\left[1\bar{1}0\right]}{H_{c}//\left[001\right]}$ ~ 0.98 near *T*_c_ and reduces monotonically to ~0.8 at the lowest temperatures, with no inversion. The same anisotropy is found in a lower *T*_c_ sample (fig. S9). We also measure *R*_ANI_(φ) in a 0.5-T in-plane (Fig. 3F) for a fixed current direction to obtain the full angular dependence of MR at *T* ≲ *T*_c_ for AlO*_x_*/KTO, and we find it to be nearly isotropic.

The *H*_c_-versus-*T*-phase diagrams for EuO*_x_*/KTO (110) and AlO*_x_*/KTO (110) are very different. Despite the highly anisotropic spin-texture anticipated for KTO (110) 2DEGs, the anisotropy observed in AlO*_x_*/KTO (110) can be explained entirely by the anisotropy of the orbital-limiting field. A square-root–like *H*_c_-versus-*T* variation for AlO*_x_*/KTO (110) is consistent with Ginzburg-Landau theory for a single band two-dimensional (2D) superconductor with an anisotropic mass tensor (*38*), where the ratio of the orbital critical field along the two in-plane directions is approximately related to the inverse ratio of the effective masses. Our resistance measurements in the normal state are consistent with an effective mass $m_{\left[1-10\right]}$ > $m_{\left[001\right]}$, resulting in *H*_c_//[001] > *H*_c_//[1$\bar{1}$0], as observed. Fits of the critical field using Werthamer-Helfand-Hohenberg (WHH) (*39*) theory for a lower *T*_c_ sample with a wider dynamic range in temperature are consistent with a reduced *g*-factor (fig. S16). While effective mass anisotropy is temperature independent, a reduced *g*-factor combined with anisotropic spin-orbit scattering can result in a modest variation of the anisotropy as a function of temperature (fig. S16).

On the other hand, the unconventional *H*_c_-versus-*T* data for EuO*_x_*/KTO suggest contributions of both orbital and spin pair-breaking effects. As noted earlier, at temperatures below *T*_c_, we do not observe hysteretic effects at fields of ~115 Oe as we did for the data in Fig. 2. Furthermore, the ferromagnetic EuO*_x_* moment saturates well below the fields where we continue to observe a strong reduction in *H*_c_ versus *T* (the “crossing point” is at *H* > 3 T, while the EuO*_x_* moment saturates at *H* < 0.4 T). Thus, we believe that the observed effects for *T* < *T*_c_ are not due to ferromagnetic EuO*_x_*. Rather, our data are reminiscent of Chevrel phase superconductors like EuMo_6_S_6_ where an internal field due to the presence of local moments has a profound impact on *H*_c_ and can give rise to an inflection-like behavior near *T*_c_ (*40*). We suggest a scenario involving paramagnetic Eu^2+^ impurity ions that diffuse into KTO (*17*, *41*, *42*) and interact via exchange interactions with the anisotropic spin texture of the KTO (110) 2DEG. The external field rapidly polarizes the Eu^2+^ spins at low temperatures, and they, in turn, interact with electrons at the Fermi surface via said exchange interaction. This gives rise to an extra pair-breaking term that acts to suppress *H*_c_ for temperatures near *T*_c_, which follows the polarization of the Eu moments. While an exchange coupling also exists between the ferromagnetic Eu^2+^ in EuO*_x_* and conduction electrons in 2DEGs, the ferromagnetic moment rapidly saturates below 0.1 T; thus, it is insufficient to support the continued rapid change of critical field in EuO*_x_*/KTO (110) for fields beyond 1 T applied along [001] near *T*_c_. We note that the external magnetic fields interact via the Zeeman effect with the total moment (*L* + 2*S*) of the electrons in the KTO (110) 2DEGs, which is strongly reduced due to the antialigned orbital and spin moments that nominally cancel. On the other hand, the exchange interaction between the Eu^2+^ impurities and the conduction electron in the KTO (110) 2DEG involves only the electron spin (*S*) and is not affected by the reduced total moment.

To illustrate this, we generalize an approximate formula given by Klemm, Luther, and Beasley (KLB) (*43*) by including an exchange field in their pair-breaking function α(h) that determines *H*_c_

$$\text{ln}\left(t\right)=\psi \left(\frac{1}{2}\right)-\psi \left[\frac{1}{2}+\frac{\alpha \left(h\right)}{2\pi k_{B}T}\right]$$ (1)

where ψ is the digamma function, *t* is the reduced temperature (*T/T*_c_), and *h* is the reduced field, which enters α(h) as *h*^2^ as we are considering in-plane fields.

$$\alpha \left(h\right)=ch^{2}+\frac{1}{2b}{\left[\left(\frac{g}{2}\right)h+h_{J}\right]}^{2}$$ (2)

where *c* is π*T*_c_, *b*^−1^ is 3τ_so_ with τ_so_ being the spin-orbit scattering time, and *h_J_* is the reduced exchange field between the paramagnetic Eu^2+^ impurity ions (*J* = 7/2) and the tantalum conduction electrons, which is described by a Brillouin function $h_{J}=h_{J0}B_{J}\left({\mathrm{Jg}}^{\prime }\mu_{B}H/k_{B}T\right)$^30^ ($g^{\prime }$= 2). The first term in α(*h*) contributes to orbital pair breaking, while the second term includes contributions from the Zeeman and exchange coupling to the spins of the conduction electrons. Here, *h*_*J*0_ is the magnitude of the exchange field depending on the microscopic exchange integrals and the concentration of Eu-ions. *h*_*J*0_ also depends on the angle between the in-plane field *h* and the [001] axis due to the anisotropy of the spin texture of the Ta-5d states. Because the spin texture in Fig. 1B has a uniaxial in-plane anisotropy, *h_J_* is larger for fields along [001] compared to fields along [1$\bar{1}$0]. Thus, when the external field is applied along the [001] direction, the pair-breaking function α(h) will rapidly increase at small fields following the *H*/*T* dependence of the Brillouin function. Due to this “boost” to α(h) provided by the exchange field, the required external field for pair-breaking is greatly reduced, and *H*_c_//[001] is much smaller for *T* near *T*_c._ Once the moment is saturated, the relative contribution of the “exchange” term is diminished as the field increases further. Therefore, with the decrease of temperature and, correspondingly, the increase in critical field, *H*_c_ versus *T*_c_ is gradually dominated by pair-breaking due to orbital effects. For this reason, both EuO*_x_*/KTO and AlO*_x_*/KTO show similar behavior at low *T*. We extract α(h) from our experimental *H*_c_ and fit it using Eq. 2 assuming *g* = 0.5 (fig. S17) and find that $\frac{h_{J}\;\left[001\right]}{h_{J}\;\left[1\bar{1}0\right]}$ ~ 1.33. An alternative fit to the data can be done by using an in-plane formula for *H*_c_, which is based on WHH supplemented by the exchange field (fig. S17) (*44*). As with the KLB fit, the exchange field has a negative sign with respect to applied field in the WHH fit, and a similar ratio of $\frac{h_{J}\;\left[001\right]}{h_{J}\;\left[1\bar{1}0\right]}$ ~ 1.29 is obtained. While a nonzero *g*-factor implies a Zeeman contribution to pair-breaking from the applied field, the exchange field remains the dominant pair-breaking factor near *T*_c_.

### Diffusion of Eu ions across the KTO (110) interface

**Fig. 4.**
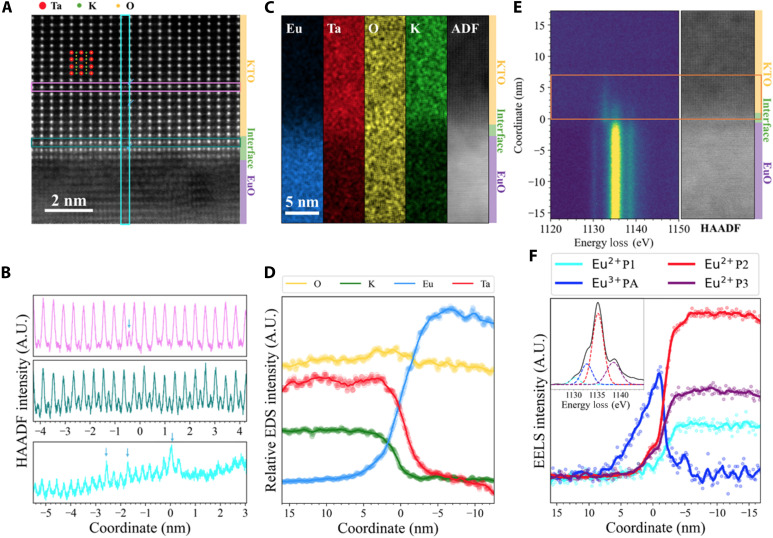
Cross section of EuO/KTO(110) interface. (**A**) Atomic resolution HAADF image of a EuO/KTO [110] interface. (**B**) Intensity line profile of the three colored lines marked in (A); the higher intensity column of atoms marks the replacement of K by Eu. (**C**) Larger field-of-view energy-dispersive x-ray spectroscopy (EDS) mapping and HAADF image across the interface. (**D**) EDS intensity profile of K, Ta, O, and Eu with respect to the vertical direction in (C) from KTO to EuO across the interface. (**E**) 2D electron energy loss spectroscopy (EELS) spectra (left) correlated to the HAADF image (right) at the EuO/KTO [110] interface. The orange box indicates the spatial extent of superconductivity, overlapping with diffused Eu^2+^/Eu^3+^ (**F**) Spatial profiles for the Eu^2+^ (1130.3, 1135.2, and 1138.5 eV) and Eu^3+^ (1132.8 eV) peaks in the EELS spectra measured across the EuO/KTO [110] interface. A selected spectrum and its decomposed peaks are shown in the inset. The zero position in (B), (D), and (F) is the EuO/KTO [110] interface. A.U., arbitrary units.

Scanning transmission electron microscopy measurements yield information on the distribution of Eu ions in the vicinity of the EuO*_x_*/KTO (110) interface, where cation interdiffusion and presence of O vacancies can lead to the formation of a 2D electron gas (*42*). Figure 4A shows a cross-sectional high-angle annular dark field (HAADF) image along the KTO [110] zone-axis orientation, where the Eu ions that have diffused into KTO are evident from the enhanced *Z*-contrast on the K sites (Fig. 4B and fig. S11), up to several atomic layers into the KTO. To obtain further insight regarding diffusion of Eu, we also analyze the interfacial composition using energy-dispersive x-ray spectroscopy (EDS) (Fig. 4C). The concentration of Eu decreases as it goes from EuO*_x_* into KTO, and the presence of Eu persists beyond 5 nm into KTO (Fig. 4D). Electron energy loss spectroscopy (EELS) of Eu also indicates diffusion of Eu into the KTO (Fig. 4E). In particular, the Eu fine structure measured using EELS reveals new satellite peaks that emerge when scanning from EuO*_x_* into the KTO (Fig. 4E). The intensity of the satellite peak at 1133 eV corresponding to Eu^3+^ rises near the interface before decaying in the KTO within 6.8 ± 1.0 nm, while the spectral signatures for Eu^2+^ peaked at 1135.2 eV decay smoothly going from EuO*_x_* into the KTO within about 3.7 ± 1.1 nm. This is within the effective thickness of the superconducting electron gas at the EuO*_x_*/KTO interface of ~7 nm (fig. S12). Thus, our findings are consistent with a mixture of Eu^2+^ and Eu^3+^ ions within KTO near the interface (Fig. 4F). The presence of Eu^2+^, which has a magnetic moment of 7$\mu_{B}$, is consistent with the field dependence of the pair-breaking parameter that we observe at temperatures just below *T*_c_. On the other hand, Eu^3+^ has zero net moment and will not respond to external fields. Thus, we hypothesize that, when an external field is applied, paramagnetic Eu^2+^ ions within the KTO align with the field and interact with the electrons in the KTO (110) 2DEG through exchange coupling. Thus, we surmise that diffusion of Eu^2+^ into KTO is central to the exchange-induced effects that we describe here, and controlling this is subject of future work.

### Anisotropic WAL for in-plane magnetic fields

**Fig. 5.**
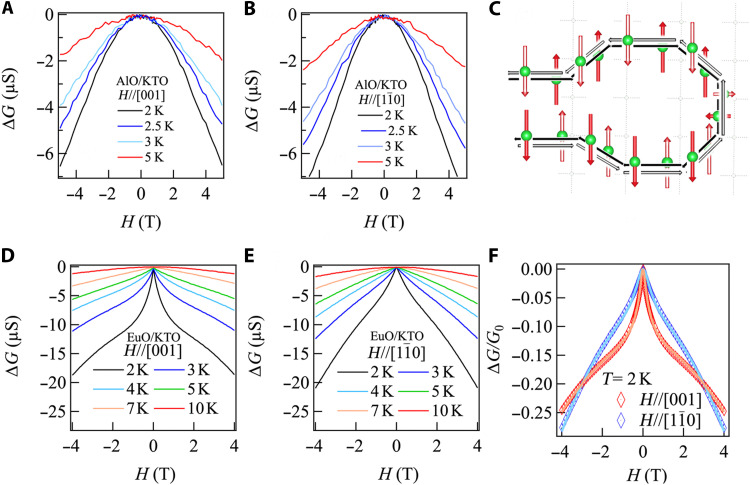
Anisotropic WAL of KTO (110). Field dependence of the magnetoconductance at different temperatures for AlO*_x_*/KTO for an in-plane magnetic field applied (**A**) along the [001] direction and (**B**) along the [1$\bar{1}$0] direction. (**D** and **E**) Same as (A) and (B), but for EuO*_x_*/KTO. (**C**) Schematic of the destructive interference for an electron scattering in time-reversed closed loop paths in KTO (110) with spins (shown by solid and hollow red arrows) locked along the [001] axis. (**F**) Weak antilocalization (WAL) fits to the magnetoconductance of EuO*_x_*/KTO at 2 K for both in-plane field directions as described in the text.

To further corroborate the uniaxial in-plane spin texture, we measure magnetoconductance at *T* > *T*_c_ for in-plane fields up to 5 T, where we find signatures of an anisotropic in-plane weak antilocalization (WAL) in EuO*_x_*/KTO (110) samples. Quantum interference of conduction electrons in the presence of SOC results in a positive magnetoresistance known as WAL. WAL for an out-of-plane field has been observed for 2DEGs in both KTO (111) and KTO (110) (*36*, *45*–*47*). However, in 2DEGs formed at the NdTiO_3_/SrTiO_3_ interface, it was shown that exchange coupling due to local magnetic moments can enhance the dephasing of electrons under in-plane fields (*48*) and that WAL effects in this geometry can probe the in-plane spin susceptibility. Thus, we first measured the magnetoconductance in AlO*_x_*/KTO (110) at different temperatures for in-plane fields along [1$\bar{1}$0] and [001] (Fig. 5, A and B). A broad quadratic field dependence of the magnetoconductance is observed along both directions, which is presumably due to Zeeman dephasing in applied in-plane fields. We observe a decrease in the magnetoconductance as the temperature increases due to the loss of phase coherence. A cusp-like behavior is only observed for fields along the out-of-plane direction (fig. S13A). This mirrors what occurs for *H*_c_, where the orbital field enters linear in *H* for perpendicular fields but quadratic in *H* for parallel fields, given that the Cooperon that is associated with WAL has similar properties to the Cooper pair propagator for superconductivity.

Figure 5 (D and E) shows the magnetoconductance measured on EuO*_x_*/KTO (110) at different temperatures for the two in-plane field directions. In strong contrast to AlO*_x_*/KTO (110), a sharp cusp in the field dependence of the magnetoconductance is observed when the field is applied along [001]. As the temperature increases from 2 K, the cusp feature is attenuated, and the magnetoconductance decreases sharply as quantum corrections are lost. For fields applied along [1$\bar{1}$0], a noticeably broader cusp-like feature is observed at 2 K, but with a similar temperature dependence as for [001]. Because the orbital corrections to the magnetoconductance should be quadratic and small for in-plane fields in a quasi-2D limit, we look to the Zeeman contribution for the dephasing of WAL in the magnetoconductance (*48*)

$$∆G\left(H_{//}\right)=-\frac{G_{0}}{2}\text{ln}\left[1+\frac{{∆}_{\Phi }\left(H_{//}\right)}{B_{\Phi }}\right]$$ (3)

where $\Delta_{\Phi }\left(H_{//}\right)=\frac{{\left[2\mu_{B}\left(H_{//}\right)\right]}^{2}}{{\left(4\mathrm{eD}\right)}^{2}B_{\text{so}}}$. Here, *D* is the diffusion constant, *B*_so_ is the field scale associated with spin-orbit scattering, $B_{\Phi }$ is the orbital dephasing field scale, and *G*_0_ is the quantum of conductance. For an in-plane field *H*, this gives rise to a quadratic field dependence. However, as with *H*_c_, for EuO*_x_*/KTO, we expect that the Eu impurity moments in the KTO 2DEGs will induce an additional exchange term that will sharply enhance the effective field that enters into the Zeeman term (*49*) *H*_//_ *=* $\frac{g}{2}$*H + H_J_*. The first term in *H*_//_ is small because *g* is strongly reduced because of quenching of the total moment, and the field-induced dephasing is dominated by the exchange interaction between Eu moments and the uniaxial half-Rashba spin-polarized electrons (Fig. 5C and fig. S13B). At low temperatures, the Eu moments align rapidly with *H*, leading to the pronounced boost in the exchange interaction at low fields with electrons in the KTO 2DEGs, and a quasilinear behavior is seen in the magnetoconductance. At higher fields, the impurity moments saturate, and we anticipate crossing over to a quadratic behavior as expected from orbital contributions. We thus fit our data using Eq. 3 with the same *g* and the same exchange field as in our *H*_c_ analysis (see table S2). These fits are excellent, indicating that our *H*_c_ and magnetoconductance analysis is consistent with one another.

## DISCUSSION

For context, the crossover in the anisotropy in *H*_c_ has been observed (*50*–*52*) in a quasi-1D superconductor K_2_Cr_3_As_3_, which arises from an anisotropic spin susceptibility along and perpendicular to its 1D chains, although these data do not show the pronounced inflection in *H*_c_ (*T*_c_) we see for fields along [001] in EuO*_x_*/KTO (110). Notably, measurements of the Knight shift in K_2_Cr_3_As_3_ in the superconducting state suggest a triplet component (*53*). Twofold modulations of the in-plane *H*_c_ have also been observed in several 2D superconductors and have been attributed to emergent nematicity (*54*) and mixing of s-wave with d-wave or p-wave order parameters (*11*, *36*). While a Lorentz force resulting from magnetic fields could lead to a twofold symmetry of the in-plane *H*_c_ if vortices move freely, the observed results in EuO*_x_*/KTO are independent of the current direction (fig. S10), which would rule out this mechanism for an anisotropic Fermi surface. More recently, twofold *H*_c_ modulations have been ascribed to anisotropic spin susceptibilities that result from 3D spin textures of the Fermi surface due to broken mirror symmetries and the interplay of parity with SOC near band-inversion points in reciprocal space (*55*–*57*). However, corroborating experimental evidence for these spin textures is not well established.

In summary, we find evidence for an in-plane uniaxial spin texture in KTO (110) 2DEGs. The texture is hidden in AlO*_x_*/KTO due to a strongly reduced *g*-factor of the Ta conduction electrons, but, for EuO*_x_*/KTO, the presence of an exchange field from the Eu ions “lights up” the Ta-*5*d spin texture and allows us to see its consequences for both the critical field in the superconducting state as well the low-temperature normal state magnetoconductance. The anisotropic response in the superconducting state is consistent with the uniaxial half-Rashba ($k_{y}{\hat{\sigma }}_{x}$) spin texture that occurs in the d*_yz_*/*d_xz_* bands. Our results also imply that carriers in d*_xy_* bands, which lack such a uniaxial spin texture, do not contribute. These carriers also do not participate in the WAL-like response to in-plane fields. The unique in-plane half-Rashba like nature of the spin texture intrinsic to KTO (110) 2DEGs may have consequences for spin-orbitronics (*58*) as the Rashba-Edelstein length should be highly anisotropic in plane. In the context of unconventional superconductivity, the triplet component of the Cooper pairs that can be admixed due to inversion symmetry breaking (*5*) should exhibit the same in-plane uniaxial anisotropy as we find here. Our results lend insight into the nature of exchange interactions at the EuO/KTO interface and the role of Eu interdiffusion in KTO. In addition to probing spin textures, magnetic proximity effects also offer a path to control superconductivity in spin-textured materials.

## MATERIALS AND METHODS

### Sample fabrication

AlO*_x_*/KTO samples were prepared by depositing pure Al on a single crystal KTO (110) substrate in a temperature range from 500° to 600°C under 10^−9^-mbar pressure in a molecular beam epitaxy chamber. The EuO*_x_*/KTO sample was prepared by depositing pure Eu on a single crystal KTO (110) substrate at a temperature of 500°C under 10^−8^-mbar oxygen pressure in a molecular beam epitaxy chamber (*17*, *19*). A 10-nm Ge capping layer was deposited on all samples as a protecting layer. Hall bar patterns were prepared using by means of a maskless laser photolithography system (MLA 150, Heidelberg; photoresist AR-P 5350, Allresist EN). Pattern transfer from the photoresist onto the samples was realized using liq-N_2_ cooled Ar-based ion-beam etching.

### Transport measurements

The low-temperature transport measurements down to 50 mK were carried out in a Bluefors dilution fridge equipped with a 9-1-1 vector magnet. The out-of-plane magnetic field component for a given in-plane magnetic field was zeroed out by searching for minima in the magnetoresistance while sweeping the out-of-plane field about zero.

The 23-mK field dependence of the resistance measurement in parallel fields was carried out in an 18-T cryogenic station at the National High Magnetic Field Laboratory, with the sample mounted on a rotator. The zeroing out of the out-of-plane component was achieved by searching for minima in the angular dependence of the resistance while rotating the sample. The magnetoconductance measurements at higher temperature were carried out through a rotator in a 14 T Physical Properties Measurement System from Quantum Design.

### Statement regarding error bars

In Figs. 1 to 3 and 5, the uncertainty/error in the plotted data points is smaller than the symbols used to represent the data. For Fig. 4, the error bars for the diffusion lengths of the Eu^2+^ and Eu^3+^ in KTO are estimated from the scatter in the intensity of the spectral signatures with position.

## Supplementary Materials

### This PDF file includes:

Supplementary Text S1 to S14 Figs. S1 to S18 Tables S1 to S3 References

## References

[1] G. Bihlmayer, P. Noël, D. V. Vyalikh, E. V. Chulkov, A. Manchon, Rashba-like physics in condensed matter. Nat. Rev. Phys. 4, 642–659 (2022).

[2] S. Picozzi, Spin–orbit coupling in quantum materials: Emergent phenomena, their modelling and examples from two-dimensional magnets. Riv. Nuovo Cimento. 47, 609–652 (2024).

[3] Y. A. Bychkov, E. I. Rashba, Properties of a 2D electron-gas with lifted spectral degeneracy. JETP Lett. 39, 78–81 (1984).

[4] M. Smidman, M. B. Salamon, H. Q. Yuan, D. F. Agterberg, Superconductivity and spin–orbit coupling in non-centrosymmetric materials: A review. Rep. Prog. Phys. 80, 036501 (2017). 28072583 10.1088/1361-6633/80/3/036501

[5] L. P. Gor'kov, E. I. Rashba, Superconducting 2D system with lifted spin degeneracy: Mixed singlet-triplet state. Phys. Rev. Lett. 87, 037004 (2001). 11461584 10.1103/PhysRevLett.87.037004

[6] J. M. Lu, O. Zheliuk, I. Leermakers, N. F. Q. Yuan, U. Zeitler, K. T. Law, J. T. Ye, Evidence for two-dimensional Ising superconductivity in gated MoS_2_. Science 350, 1353–1357 (2015). 26563134 10.1126/science.aab2277

[7] Y. Saito, Y. Nakamura, M. S. Bahramy, Y. Kohama, J. Ye, Y. Kasahara, Y. Nakagawa, M. Onga, M. Tokunaga, T. Nojima, Y. Yanase, Y. Iwasa, Superconductivity protected by spin–valley locking in ion-gated MoS_2_. Nat. Phys. 12, 144–149 (2016).

[8] J. Falson, Y. Xu, M. Liao, Y. Zang, K. Zhu, C. Wang, Z. Zhang, H. Liu, W. Duan, K. He, H. Liu, J. H. Smet, D. Zhang, Q.-K. Xue, Type-II Ising pairing in few-layer stanene. Science 367, 1454–1457 (2020). 32165427 10.1126/science.aax3873

[9] B. T. Zhou, N. F. Q. Yuan, H. L. Jiang, K. T. Law, Ising superconductivity and Majorana fermions in transition-metal dichalcogenides. Phys. Rev. B 93, 180501 (2016).

[10] K. Matano, M. Kriener, K. Segawa, Y. Ando, G. Q. Zheng, Spin-rotation symmetry breaking in the superconducting state of Cu_x_Bi_2_Se_3_. Nat. Phys. 12, 852–854 (2016).

[11] A. Hamill, B. Heischmidt, E. Sohn, D. Shaffer, K. T. Tsai, X. Zhang, X. X. Xi, A. Suslov, H. Berger, L. Forró, F. J. Burnell, J. Shan, K. F. Mak, R. M. Fernandes, K. Wang, V. S. Pribiag, Two-fold symmetric superconductivity in few-layer NbSe_2_. Nat. Phys. 17, 949–954 (2021).

[12] A. Johansson, J. Henk, I. Mertig, Theoretical aspects of the Edelstein effect for anisotropic two-dimensional electron gas and topological insulators. Phys. Rev. B 93, 195440 (2016).

[13] S. M. Frolov, M. J. Manfra, J. D. Sau, Topological superconductivity in hybrid devices. Nat. Phys. 16, 718–724 (2020).

[14] M. M. Desjardins, L. C. Contamin, M. R. Delbecq, M. C. Dartiailh, L. E. Bruhat, T. Cubaynes, J. J. Viennot, F. Mallet, S. Rohart, A. Thiaville, A. Cottet, T. Kontos, Synthetic spin-orbit interaction for Majorana devices. Nat. Mater. 18, 1060–1064 (2019). 31427741 10.1038/s41563-019-0457-6

[15] J. Linder, J. W. A. Robinson, Superconducting spintronics. Nat. Phys. 11, 307–315 (2015).

[16] M. Amundsen, J. Linder, J. W. A. Robinson, I. Zutic, N. Banerjee, Spin-orbit effects in superconducting hybrid structures. Rev. Mod. Phys. 96, 021003 (2024).

[17] C. Liu, X. Yan, D. Jin, Y. Ma, H.-W. Hsiao, Y. Lin, T. M. Bretz-Sullivan, X. Zhou, J. Pearson, B. Fisher, J. S. Jiang, W. Han, J.-M. Zuo, J. Wen, D. D. Fong, J. Sun, H. Zhou, A. Bhattacharya, Two-dimensional superconductivity and anisotropic transport at KTaO_3_ (111) interfaces. Science 371, 716–721 (2021). 33479119 10.1126/science.aba5511

[18] Z. Chen, Z. R. Liu, Y. Q. Sun, X. X. Chen, Y. Liu, H. Zhang, H. K. Li, M. Zhang, S. Y. Hong, T. S. Ren, C. Zhang, H. Tian, Y. Zhou, J. R. Sun, Y. W. Xie, Two-dimensional superconductivity at the LaAlO_3_/KTaO_3_ (110) heterointerface. Phys. Rev. Lett. 126, 026802 (2021). 33512194 10.1103/PhysRevLett.126.026802

[19] C. Liu, X. Zhou, D. Hong, B. Fisher, H. Zheng, J. Pearson, J. S. Jiang, D. Jin, M. R. Norman, A. Bhattacharya, Tunable superconductivity and its origin at KTaO_3_ interfaces. Nat. Commun. 14, 951 (2023). 36806127 10.1038/s41467-023-36309-2 PMC9941122

[20] E. A. Martínez, J. Dai, M. Tallarida, N. M. Nemes, F. Y. Bruno, Anisotropic electronic structure of the 2D electron gas at the AlO*_x_*/KTaO_3_(110) interface. Adv. Electron. Mater. 9, 2300267 (2023).

[21] P. W. Krantz, A. Tyner, P. Goswami, V. Chandrasekhar, Nonlinear hall effect in KTaO_3_ two-dimensional electron gases. arXiv:2411.09161 (2024).

[22] S. J. Poage, X. S. Gao, M. Baksi, S. Salmani-Rezaie, D. A. Muller, D. P. Kumah, C. N. Lau, J. Lorenzana, M. N. Gastiasoro, K. Ahadi, Violation of the Pauli limit at KTaO (110) interfaces. Phys. Rev. B 111, 214506 (2025).

[23] F. Y. Bruno, S. M. Walker, S. Riccò, A. de la Torre, Z. M. Wang, A. Tamai, T. K. Kim, M. Hoesch, M. S. Bahramy, F. Baumberger, Band structure and spin-orbital texture of the (111)-KTaO_3_ 2D electron gas. Adv. Electron Mater. 5, 1800860 (2019).

[24] S. Varotto, A. Johansson, B. Göbel, L. M. Vicente-Arche, S. Mallik, J. Bréhin, R. Salazar, F. Bertran, P. Le Fèvre, N. Bergeal, J. Rault, I. Mertig, M. Bibes, Direct visualization of Rashba-split bands and spin/orbital-charge interconversion at KTaO_3_ interfaces. Nat. Commun. 13, 6165 (2022). 36257940 10.1038/s41467-022-33621-1 PMC9579156

[25] S. Sugano, Y. Tanabe, H. Kamimura, *Multiplets of Transition-Metal Ions in Crystals* (Elsevier Science, 1970).

[26] A. H. Al-Tawhid, S. J. Poage, S. Salmani-Rezaie, A. Gonzalez, S. Chikara, D. A. Muller, D. Kumah, M. N. Gastiasoro, J. Lorenzana, K. Ahadi, Enhanced critical field of superconductivity at an oxide interface. Nano Lett. 23, 6944–6950 (2023). 37498750 10.1021/acs.nanolett.3c01571

[27] G. Singh, A. Jouan, G. Herranz, M. Scigaj, F. Sánchez, L. Benfatto, S. Caprara, M. Grilli, G. Saiz, F. Couëdo, C. Feuillet-Palma, J. Lesueur, N. Bergeal, Gap suppression at a Lifshitz transition in a multi-condensate superconductor. Nat. Mater. 18, 948–954 (2019). 31086324 10.1038/s41563-019-0354-z

[28] A. Annadi, Q. Zhang, X. Renshaw Wang, N. Tuzla, K. Gopinadhan, W. M. Lü, A. Roy Barman, Z. Q. Liu, A. Srivastava, S. Saha, Y. L. Zhao, S. W. Zeng, S. Dhar, E. Olsson, B. Gu, S. Yunoki, S. Maekawa, H. Hilgenkamp, T. Venkatesan, Ariando, Anisotropic two-dimensional electron gas at the LaAlO_3_/SrTiO_3_ (110) interface. Nat.Commun. 4, 1838 (2013). 23673623 10.1038/ncomms2804 PMC3674248

[29] Z. Wang, Z. Zhong, X. Hao, S. Gerhold, B. Stöger, M. Schmid, J. Sánchez-Barriga, A. Varykhalov, C. Franchini, K. Held, U. Diebold, Anisotropic two-dimensional electron gas at SrTiO_3_ (110). Proc. Natl. Acad. Sci. U.S.A. 111, 3933–3937 (2014). 24591596 10.1073/pnas.1318304111 PMC3964063

[30] P. Moetakef, J. R. Williams, D. G. Ouellette, A. P. Kajdos, D. Goldhaber-Gordon, S. J. Allen, S. Stemmer, Carrier-controlled ferromagnetism in SrTiO_3_. Phys. Rev. X 2, 021014 (2012).

[31] D. A. Dikin, M. Mehta, C. W. Bark, C. M. Folkman, C. B. Eom, V. Chandrasekhar, Coexistence of superconductivity and ferromagnetism in two dimensions. Phys. Rev. Lett. 107, 056802 (2011). 21867087 10.1103/PhysRevLett.107.056802

[32] F. Wen, Y. Cao, X. Liu, B. Pal, S. Middey, M. Kareev, J. Chakhalian, Evolution of ferromagnetism in two-dimensional electron gas of LaTiO_3_/SrTiO_3_. Appl. Phys. Lett. 112, 122405 (2018).

[33] P. W. Krantz, A. Tyner, P. Goswami, V. Chandrasekhar, Intrinsic magnetism in KTaO_3_ heterostructures. Appl. Phys. Lett. 124, 093102 (2024).

[34] X. Hua, Z. Zeng, F. Meng, H. Yao, Z. Huang, X. Long, Z. Li, Y. Wang, Z. Wang, T. Wu, Z. Weng, Y. Wang, Z. Liu, Z. Xiang, X. Chen, Superconducting stripes induced by ferromagnetic proximity in an oxide heterostructure. Nat. Phys. 20, 957–963 (2024).

[35] K. Maki, T. Tsuneto, Pauli paramagnetism and superconducting state. Prog. Theor. Phys. 31, 945–956 (1964).

[36] Z. Zhang, W. Jiang, T. Shao, Y. Qiao, X. Chen, Q. Zhao, M. Chen, R. Dou, C. Xiong, J. Nie, A spin–orbit scattering–enhanced high upper critical field at the LaAlO_3_/KTaO_3_(111) superconducting interface. New J. Phys. 25, 023023 (2023).

[37] E. G. Arnault, A. H. Al-Tawhid, S. Salmani-Rezaie, D. A. Muller, D. P. Kumah, M. S. Bahramy, G. Finkelstein, K. Ahadi, Anisotropic superconductivity at KTaO_3_ (111) interfaces. Sci. Adv. 9, eadf1414 (2023). 36791191 10.1126/sciadv.adf1414 PMC9931206

[38] M. Tinkham, *Introduction to Superconductivity* (Dover Publications, 2004).

[39] N. R. Werthamer, E. Helfand, P. C. Hohenberg, Temperature and purity dependence of the superconducting critical field, H_c2_. III. Electron spin and spin-orbit effects. Phys. Rev. 147, 295–302 (1966).

[40] O. Fischer, M. Decroux, S. Roth, R. Chevrel, M. Sergent, Compensation of the paramagnetic effect on H_c2_ by magnetic moments: 700 kG superconductors. J. Phys. C. Solid Stat. Phys. 8, L474–L477 (1975).

[41] H. Xu, H. Li, N. Gauquelin, X. Chen, W.-F. Wu, Y. Zhao, L. Si, D. Tian, L. Li, Y. Gan, S. Qi, M. Li, F. Hu, J. Sun, D. Jannis, P. Yu, G. Chen, Z. Zhong, M. Radovic, J. Verbeeck, Y. Chen, B. Shen, Giant tunability of rashba splitting at cation-exchanged polar oxide interfaces by selective orbital hybridization. Adv. Mater. 36, e2313297 (2024). 38475975 10.1002/adma.202313297

[42] B. Lama, E. Y. Tsymbal, T. R. Paudel, Effects of intermixing and oxygen vacancies on a two-dimensional electron gas at the polar TbScO_3_/KTaO_3_ (001) interface. Phys. Rev. Mater. 7, 026201 (2023).

[43] R. A. Klemm, A. Luther, M. R. Beasley, Theory of upper critical-field in layered superconductors. Phys. Rev. B 12, 877–891 (1975).

[44] O. H. Fischer, Properties of high-field superconductors containing localized magnetic moments. Helv. Phys. Acta 45, 331–397 (1972).

[45] Y. Gan, F. Yang, L. Kong, X. Chen, H. Xu, J. Zhao, G. Li, Y. Zhao, L. Yan, Z. Zhong, Y. Chen, H. Ding, Light-induced giant rashba spin–orbit coupling at superconducting KTaO_3_ (110) heterointerfaces. Adv. Mater. 35, e2300582 (2023). 36972144 10.1002/adma.202300582

[46] A. H. Al-Tawhid, J. Kanter, M. Hatefipour, D. P. Kumah, J. Shabani, K. Ahadi, Superconductivity and weak anti-localization at KTaO_3_ (111) interfaces. J. Electron. Mater. 51, 6305–6309 (2022).

[47] X. Hua, F. Meng, Z. Huang, Z. Li, S. Wang, B. Ge, Z. Xiang, X. Chen, Tunable two-dimensional superconductivity and spin-orbit coupling at the EuO/KTaO_3_(110) interface. NPJ Quant. Mater. 7, 97 (2022).

[48] X. Cai, Y. Ayino, J. Yue, P. Xu, B. Jalan, V. S. Pribiag, Disentangling spin-orbit coupling and local magnetism in a quasi-two-dimensional electron system. Phys. Rev. B 100, 081402 (2019).

[49] S. Maekawa, H. Fukuyama, Magnetoresistance in two-dimensional disordered systems: Effects of zeeman splitting and spin-orbit scattering. J. Physical Soc. Japan 50, 2516–2524 (1981).

[50] F. F. Balakirev, T. Kong, M. Jaime, R. D. McDonald, C. H. Mielke, A. Gurevich, P. C. Canfield, S. L. Bud'ko, Anisotropy reversal of the upper critical field at low temperatures and spin-locked superconductivity in K_2_Cr_3_As_3_. Phys. Rev. B 91, 220505 (2015).

[51] J. K. Bao, J. Y. Liu, C. W. Ma, Z. H. Meng, Z. T. Tang, Y. L. Sun, H. F. Zhai, H. Jiang, H. Bai, C. M. Feng, Z. A. Xu, G. H. Cao, Superconductivity in quasi-one-dimensional K_2_Cr_3_As_3_ with significant electron correlations. Phys. Rev. X 5, 011013 (2015).

[52] H. K. Zuo, J. K. Bao, Y. Liu, J. H. Wang, Z. Jin, Z. C. Xia, L. Li, Z. Xu, J. Kang, Z. W. Zhu, G. H. Cao, Temperature and angular dependence of the upper critical field in K_2_Cr_3_As_3_. Phys. Rev. B 95, 014502 (2017).

[53] J. Yang, J. Luo, C. Yi, Y. Shi, Y. Zhou, G. Q. Zheng, Spin-triplet superconductivity in K_2_Cr_3_As_3_. Sci. Adv. 7, eabl4432 (2021). 34936458 10.1126/sciadv.abl4432 PMC8694604

[54] I. Silber, S. Mathimalar, I. Mangel, A. K. Nayak, O. Green, N. Avraham, H. Beidenkopf, I. Feldman, A. Kanigel, A. Klein, M. Goldstein, A. Banerjee, E. Sela, Y. Dagan, Two-component nematic superconductivity in 4Hb-TaS_2_. Nat. Commun. 15, 824 (2024). 38280890 10.1038/s41467-024-45169-3 PMC10821864

[55] Y. M. Xie, B. T. Zhou, K. T. Law, Spin-orbit-parity-coupled superconductivity in topological monolayer WTe_2_. Phys. Rev. Lett. 125, 107001 (2020). 32955301 10.1103/PhysRevLett.125.107001

[56] J. Cui, P. Li, J. Zhou, W. Y. He, X. Huang, J. Yi, J. Fan, Z. Ji, X. Jing, F. Qu, Z. G. Cheng, C. Yang, L. Lu, K. Suenaga, J. Liu, K. T. Law, J. Lin, Z. Liu, G. Liu, Transport evidence of asymmetric spin-orbit coupling in few-layer superconducting 1*T*_d_-MoTe_2_. Nat. Commun. 10, 2044 (2019). 31053717 10.1038/s41467-019-09995-0 PMC6499809

[57] E. Z. Zhang, Y. M. Xie, Y. Q. Fang, J. L. Zhang, X. Xu, Y. C. Zou, P. L. Leng, X. J. Gao, Y. Zhang, L. F. Ai, Y. D. Zhang, Z. H. Jia, S. S. Liu, J. Y. Yan, W. Zhao, S. J. Haigh, X. F. Kou, J. S. Yang, F. Q. Huang, K. T. Law, F. X. Xiu, S. M. Dong, Spin-orbit-parity coupled superconductivity in atomically thin 2M-WS_2_. Nat. Phys. 19, 106–113 (2023).

[58] P. He, S. M. Walker, S. S. L. Zhang, F. Y. Bruno, M. S. Bahramy, J. M. Lee, R. Ramaswamy, K. Cai, O. Heinonen, G. Vignale, F. Baumberger, H. Yang, Observation of out-of-plane spin texture in a SrTiO_3_ (111) two-dimensional electron gas. Phys. Rev. Lett. 120, 266802 (2018). 30004757 10.1103/PhysRevLett.120.266802

[59] H. Zhang, Y. Yun, X. Zhang, H. Zhang, Y. Ma, X. Yan, F. Wang, G. Li, R. Li, T. Khan, Y. Chen, W. Liu, F. Hu, B. Liu, B. Shen, W. Han, J. Sun, High-mobility spin-polarized two-dimensional electron gases at EuO/KTaO_3_ interfaces. Phys. Rev. Lett. 121, 116803 (2018). 30265094 10.1103/PhysRevLett.121.116803

[60] T. Yamasaki, K. Ueno, A. Tsukazaki, T. Fukumura, M. Kawasaki, Observation of anomalous Hall effect in EuO epitaxial thin films grown by a pulse laser deposition. Appl. Phys. Lett. 98, 082116 (2011).

[61] D. Xiao, W. Zhu, Y. Ran, N. Nagaosa, S. Okamoto, Interface engineering of quantum Hall effects in digital transition metal oxide heterostructures. Nat. Commun. 2, 596 (2011). 22186892 10.1038/ncomms1602

[62] A. Jain, S. P. Ong, G. Hautier, W. Chen, W. D. Richards, S. Dacek, S. Cholia, D. Gunter, D. Skinner, G. Ceder, K. A. Persson, Commentary: The materials project: A materials genome approach to accelerating materials innovation. APL Materials 1, 011002 (2013).

[63] W. T. Vetterling, S. A. Teukolsky, W. H. Press, B. P. Flannery, *Numerical Recipes* (Cambridge Univ. Press, ed. 2, 1992).

[64] V. Jaccarino, M. Peter, Ultra-high-field superconductivity. Phys. Rev. Lett. 9, 290–292 (1962).

