# Supplementary material for: Uniaxial spin texture in a superconducting electron gas revealed by exchange interactions
Source: Sci Adv. 2026 May 8;12(19):eaeb1601. doi: 10.1126/sciadv.aeb1601 (PMC13155338; doi:10.1126/sciadv.aeb1601)
Supplement: Supplementary file 1 — Supplementary Text S1 to S14 Figs. S1 to S18 Tables S1 to S3 References [file sciadv.aeb1601_sm.pdf]

Science Advances
AAAS

Supplementary Materials for

Uniaxial spin texture in a superconducting electron gas revealed by exchange interactions

Junyi Yang *et al.*

Corresponding author: Anand Bhattacharya, anand@anl.gov

Sci. Adv. **12**, eaeb1601 (2026)
DOI: 10.1126/sciadv.aeb1601

This PDF file includes:

Supplementary Text S1 to S14
Figs. S1 to S18
Tables S1 to S3
References

S1: Patterned 2DEG KTO devices

Hall bar devices were fabricated on a KTO (110) sample along different crystal axes. A square device was patterned in the center for a Van der Pauw geometry measurement (Figure S1).

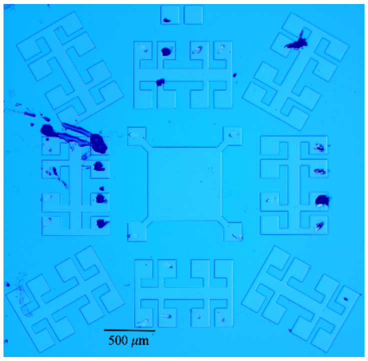

Figure S1. Devices fabricated on KTO (110) under an optical microscope. The black dots are wire bonder residuals after measurements.

S2: XRD measurement for in-plane orientations

The patterned thin film sample was mounted on the dilution fridge puck and the puck was mounted on the sample stage of a X'Pert X-ray diffractometer (Fig. S2 (a)). The $\theta - 2\theta$ measurement was first carried out along the out-of-plane direction where a clear [110] KTO Bragg peak was observed (Fig. S2 (b)). Due to the absence of a monochromator on the diffractometer, Bragg diffractions

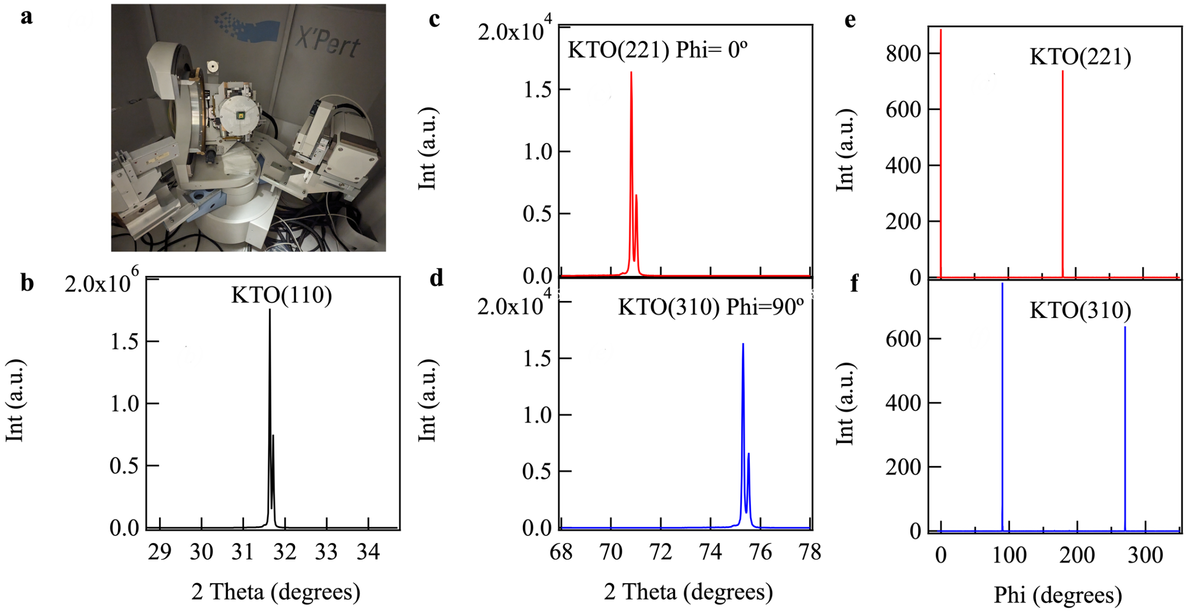

Figure S2. (a) Schematic of the XRD measurement setup. (b) $\theta - 2\theta$ scan around KTO (110). (c) $\theta - 2\theta$ scan around KTO (221). (d) Phi dependence of the intensity of KTO (221). (e) $\theta - 2\theta$ scan around KTO (310). (f) Phi dependence of the intensity of KTO (310).

due to both Cu K-α and Cu K-β were present. To resolve the in-plane orientation of the sample, a survey of different diffraction peaks was conducted. The Bragg peak [221], which is equivalent to [001]+2[110], was observed at Phi = 0° (Fig. S2 (c)). The Phi dependence of the Bragg peak [221] shows that it is only observed for Phi = 0° and 180° (Fig. S2 (d)). The peak intensities at Phi = 0 and 180° differ because of slight off-axis alignment. On the other hand, the Bragg peak [310], which is equivalent to [1-10]+2[110], is observed at Phi = 90° (Fig. S2 (e)). The Phi dependence of the Bragg peak [310] shows that it is only observed at Phi = 90° and 270° (Fig. S2 (f)). To avoid confusion of orientation in transport measurements, the sample was kept on the same puck and directly mounted on to the dilution fridge.

S3: Magnetoresistance for EuO_x/KTO around the EuO magnetic transition

The field dependence of the magnetoresistance for EuO_x/KTO for temperatures in the range of 50-80 K is shown in Fig. S3 (a) with H // [001] (in plane). The field dependence of the magnetoresistance at low fields is quadratic for a temperature (80 K) above the Curie temperature of EuO, and it becomes quasi-linear for temperatures below this transition. The temperature dependence of the magnetoresistance is shown in Fig. S3 (b). The magnetoresistance is defined as [R(-3T)-R(0T)]/R(0T) and it is negative for all measured temperatures. While a change in slope is observed close to the Curie temperature of EuO, the overall magnetoresistance is less than 0.1%. While the small negative magnetoresistance is consistent with a previous report on a EuO/KTO (001) interfacial electron gas (*59*), we also note that we do not observe any evidence for an insulator-metal transition in our EuO_x overlayer as has been observed in EuO thin films (*60*).

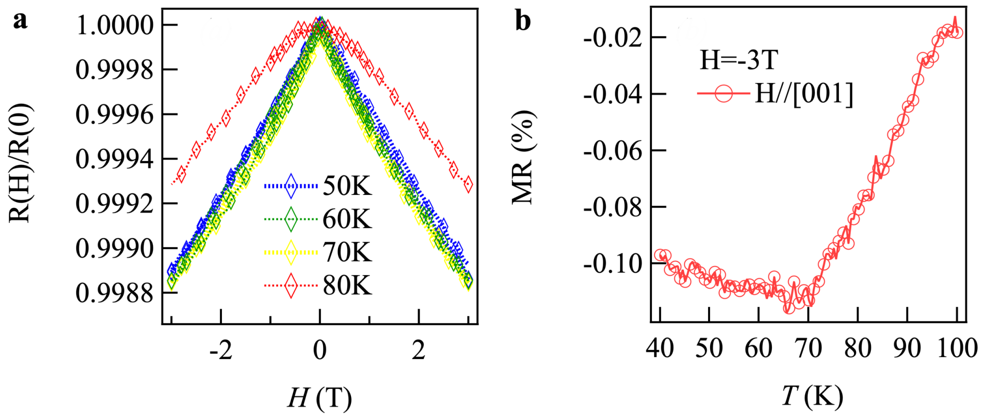

Figure S3 (a) Field dependence of the resistance of EuO_x/KTO at various temperatures for H//[001]. (b) Temperature dependence of the magnetoresistance of EuO_x/KTO for a -3T field. Note the anomaly at the Curie temperature of EuO.

S4: Field dependence of the resistance of EuO_x/KTO

The field dependence of the resistance of EuO_x/KTO at 23 mK is shown in Fig. S4. The field is applied along [001]/[1-10] respectively.

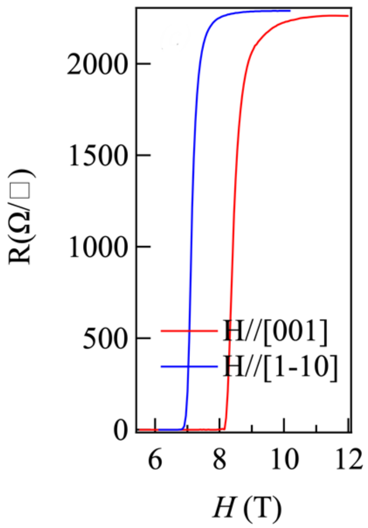

Figure S4. Field dependence of the resistance of EuO_x/KTO(110) with a field along [001] (red) and [1-10] (blue).

S5&S6: In-plane anisotropy for EuO_x/KTO samples with T_c = 1050 mK and T_c = 890 mK

The temperature dependence of the resistance under in-plane fields along [001] and [1-10] is shown in Fig. S5 (a) and (b). The transition temperature is about 890 mK. The critical field vs temperature is extracted from Fig. S5 (a) and (b) and presented in Fig. S5 (c). The critical field is larger along [001] at low temperatures, while the critical field along [1-10] is larger at temperatures close to T_c. A similar measurement is carried out for sample with T_c = 1050 mK.

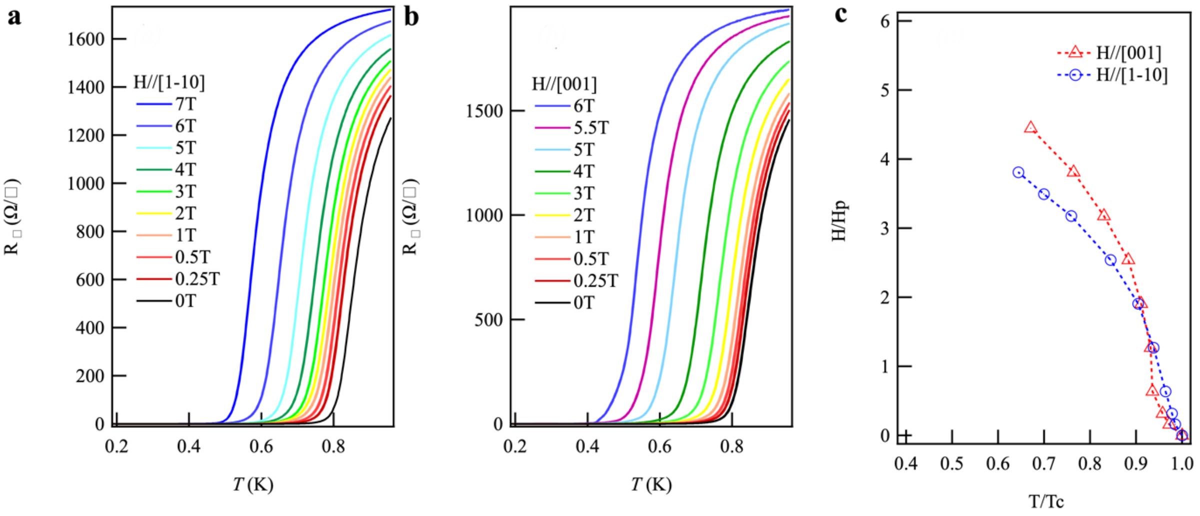

Figure S5. EuO_x/KTO sample with T_c = 890 mK (a) Temperature dependence of the resistance for different H//[001]. (b) Temperature dependence of the resistance for different H//[1-10]. (c) Critical field vs critical temperature for the two field directions.

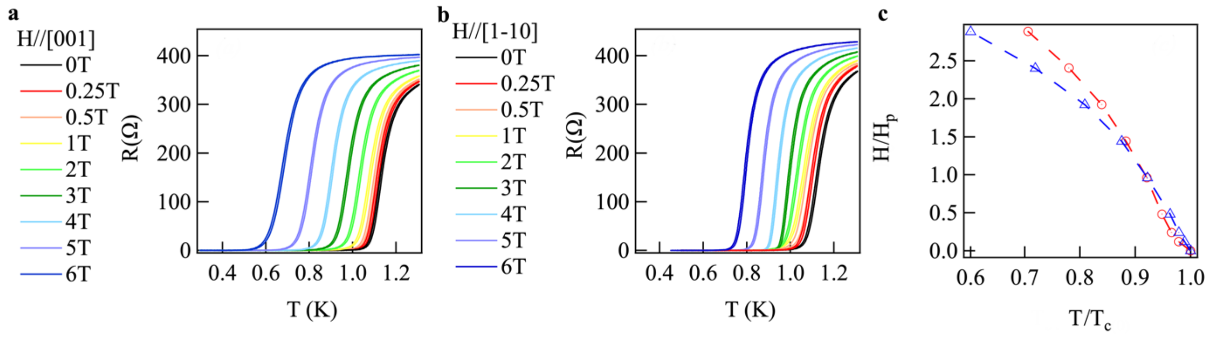

Figure S6. EuO_x/KTO sample with T_c = 1050 mK Temperature dependence of the resistance for different H//[001]. (b) Temperature dependence of the resistance f different H//[1-10]. (c) Critical field vs critical temperature for the two field directions.

S7: In-plane anisotropy for a EuO_x/KTO sample with T_c = 275 mK

The temperature dependence of the resistance under in-plane fields along [001] and [1-10] is shown in Fig. S7 (a) and (b). The transition temperature is about 275 mK. The critical field vs critical temperature is extracted from Fig. S7 (a) and (b) and presented in Fig. S7 (c). The critical field is larger along [001] at low temperatures, while the critical field along [1-10] is larger at temperatures close to T_c. The angular dependence of the resistivity with respect to the in-plane field direction at a temperature close to T_c is presented in Fig. S7 (d). The resistance for a field along [1-10] is smaller than that along [001], which is consistent with the higher T_c EuO_x/KTO sample presented in the main text. While the anisotropic behavior remains for this lower T_c EuO_x/KTO sample, its anisotropy is smaller.

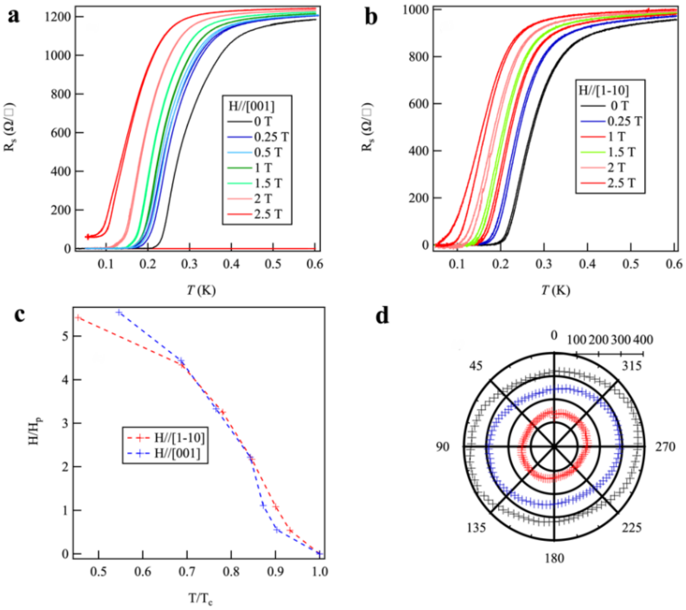

Figure S7. (a) Temperature dependence of the resistance for different H//[1-10] for EuO_x/KTO with T_c~ 275 mK. (b) Temperature dependence of the resistance for different H//[001]. (c) Critical field vs critical temperature for the two field directions. (d) Angular dependence of the resistance versus the in-plane field direction at 255 mK. The black curve denotes the field of 0.5T, the blue curve denotes the field of 0.25T and the red curve denotes the field of 0.1T.

S8: In-plane anisotropy for AlO_x/KTO sample with $T_c = 1080$ mK

The temperature dependence of the resistance under in-plane fields along [001] and [1-10] is shown in Fig. S8 (a) and (b).

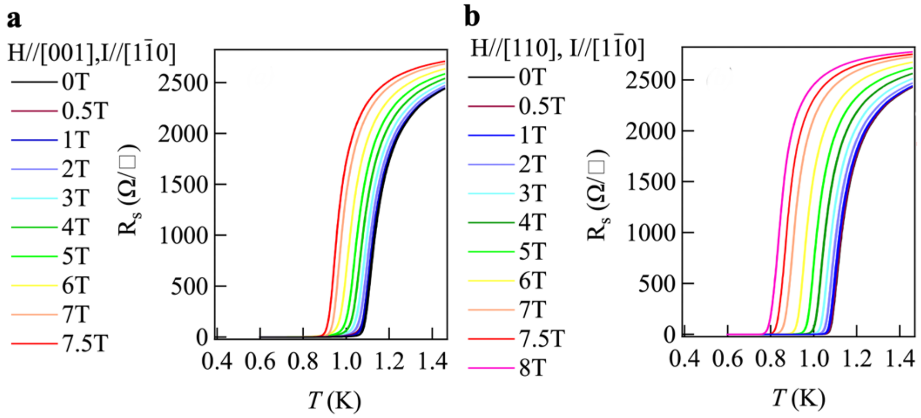

Figure S8. AlO_x/KTO sample with T_c = 1080 mK Temperature dependence of the resistance for different H//[001]. (b) Temperature dependence of the resistance for different H//[1-10].

S9: In-plane anisotropy for AlO_x/KTO sample with $T_c = 575$ mK

The temperature dependence of the resistance for in-plane fields along [001] and [1-10] are shown in Fig. S9 (a) and (b). The transition temperature is about 575 mK. The critical field vs temperature is extracted from Fig. S9 (a) and (b) and presented in Fig. S9 (c). The critical field is larger for fields along [001] compared to [1-10], displaying the characteristic square-root dependence for a 2D superconductor in a parallel field.

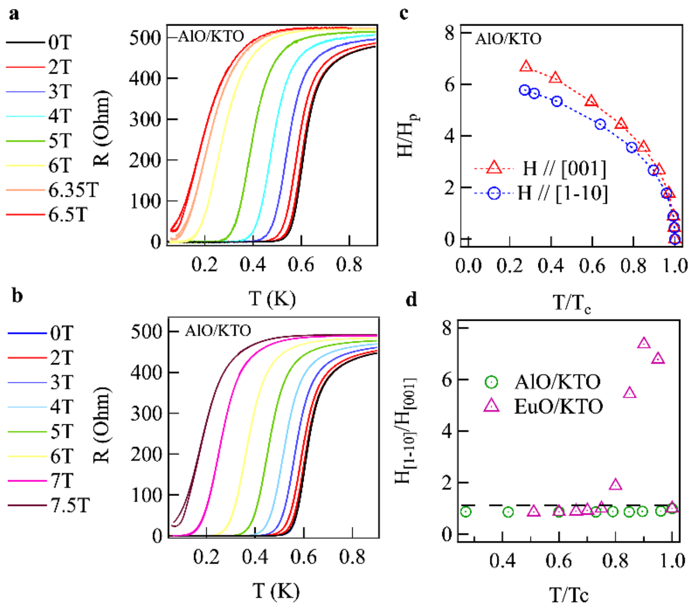

Figure S9 (a) Temperature dependence of the resistance for different H//[1-10] and (b) for different H//[001]. (c) Critical field vs critical temperature for AlO_x/KTO with a T_c~ 575 mK for the two field directions. (d) $\frac{H_c\,//\,[1\bar{1}0]}{H_c\,//\,[001]}$ for AlO_x/KTO, compared with EuO_x/KTO (110) from Fig. 3 in the main text.

S10: Angular dependence of the resistance for different current directions

Figure S10 presents the angular dependence of the resistance along different field directions for two in-plane current directions near T_c at two values of H. The results show that the anisotropic behavior for EuO_x/KTO close to T_c is independent of the current direction.

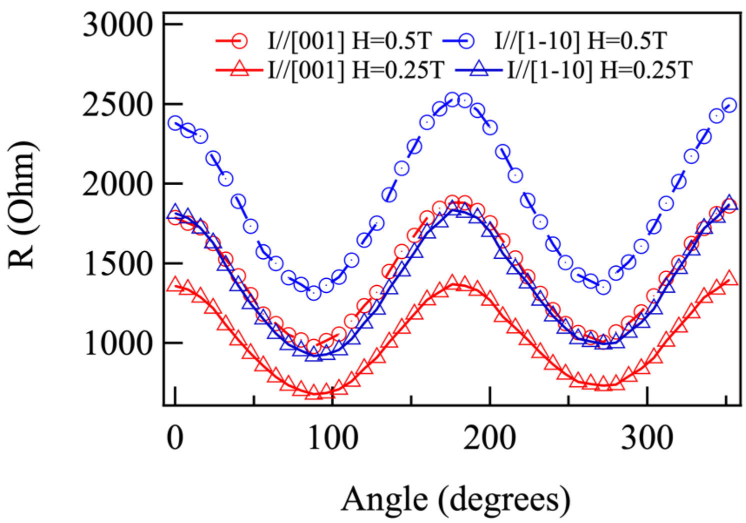

Figure S*10* Angular dependence of the resistance at 840 mK with respect to the in-plane field direction for two different values of H for EuO_x/KTO(110). Red denotes the current along [001] and blue denotes the current along [1-10].

S11: Characterizing the EuO_x/KTO (110) interface with TEM

The cross-sectional sample was prepared using a focused-ion beam (FIB, model Helios Hydra 5, FEI, USA). The atomic resolution STEM imaging, EDS and EELS, as presented in Figure. 4 and Figure. S11, were conducted on a Spectra 200 TEM (Thermo-Fisher, USA) with a cold field-emission electron source. The microscope operational voltage is at 200 kV. STEM imaging was performed using a HAADF detector with a probe convergence angle of 21.4 mrad and the camera length of 62 mm. The STEM dwell time was 200 ns with 1.29 s per frame. EDS mapping was performed to determine the chemical composition at the interface of EuO/KTO. The EDS spectra were acquired, using a four-quadrant Super-X detector (Thermo-Fisher Scientific) with the acquisition time of 50 µs per spectrum. We collect the EDS spectra in STEM mode for Figure. 4(c-d) over a sample area of 42 x 42 nm^2 and for Figure. S11 over an area of 3.4 x 6.7 nm^2. The EDS mappings were originally recorded as 60-frame stacks. The electron beam current was kept at 70 pA for both figures. A sample thickness of 40 nm was measured by zero and low loss EELS log ratio. The EELS spectra in Figure. 4(e-f) were collected using a Gatan Image Filter Quantum ER system. EELS spectra were fitted by matrix inversion deconvolution as implemented in Figure. 4(f). Per our estimates, the concentration of Eu quickly drops from ~ 10% of the K/O column (~ 20% of K sites substituted by Eu) right near EuO/KTO interface to 3% (6% of K sites substituted by EuO) at a depth of 2 nm into $KTaO_3$. It is worth noting that due to channeling effects, our estimated Eu concentration can be regarded as a semi-quantitative lower bound under the fitting and imaging conditions used.

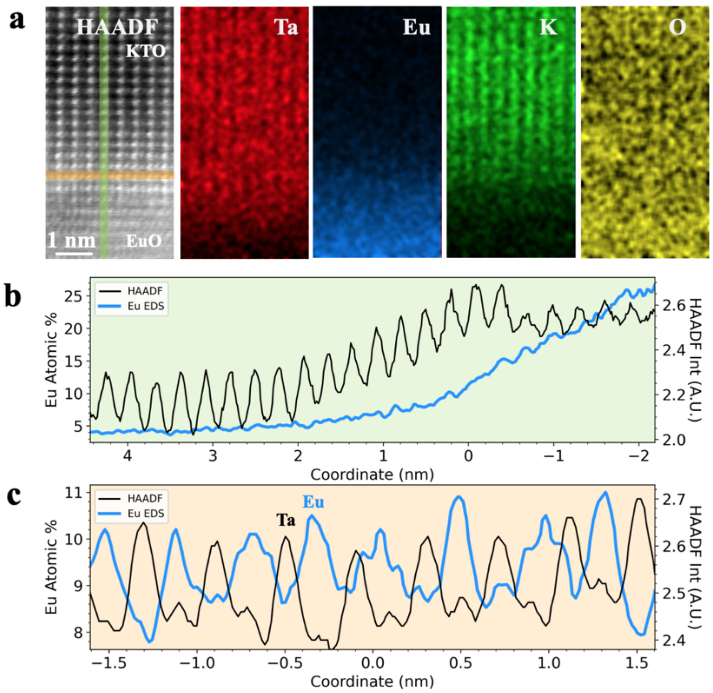

Figure S*11* a) High resolution STEM-EDS images of a EuO/KTO[110] interface. b) A vertical line profile of Eu atomic percentage from L and M peak plotted together with the HAADF intensity across the interface marked with a green color line in a). c) A horizontal line profile of Eu atomic percentage from L and M peak and the HAADF intensity at the interface as marked with an orange color line in a). The image size in a) is 3.4 x 6.7 nm^2.

S12: Out-of-plane critical field

The temperature dependence of the resistance under different out-of-plane fields was measured for a EuO_x/KTO sample (Fig. S12 (a)). The critical field for out-of-plane fields is significantly smaller compared to the critical field for in-plane fields, as expected given the 2D nature of the superconductivity of the 2DEGs. The critical field vs critical temperature was extracted from Fig. S12(a) and presented in Fig. S12 (b). The Ginzburg-Landau model for a thin film with the field out of-plane gives $H_c = \frac{\Phi_0}{2\pi\xi^2}(1-\frac{T}{T_c})$. The coherence length ξ is extracted from Fig. S8 (b) and found to be 20.8 nm which is consistent with previous results. The effective thickness of superconductivity state can be extracted from $H_{c,//} = \frac{\Phi_0}{2\pi\xi d}\sqrt{[12(1-\frac{T}{T_c})]}$. Using the critical field we measured at the lowest temperature in Fig. 3S, we obtain the effective thickness of 6.5 nm (7.6 nm) for H//[001] (H//[1-10])

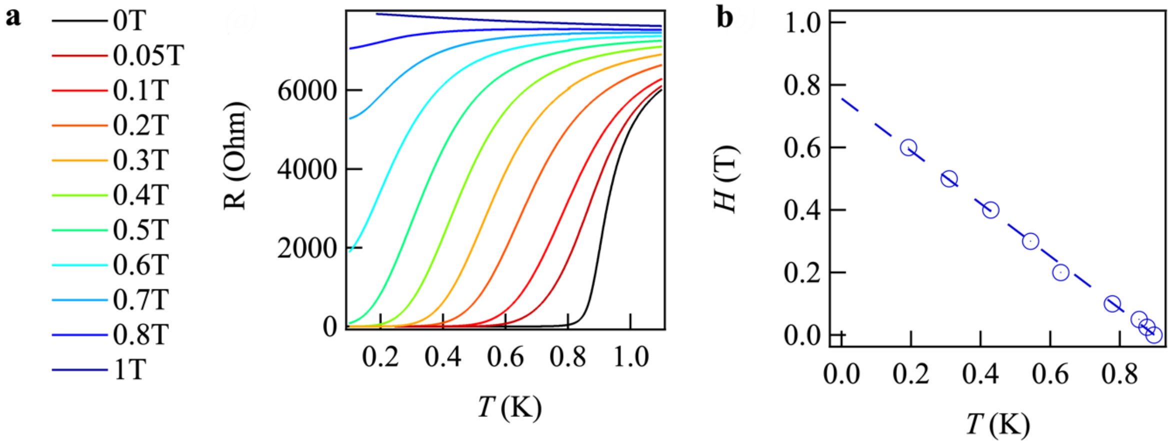

Figure S12 (a) Temperature dependence of the resistance for different out-of-plane fields for EuO_x/KTO(110). (b) Critical field vs critical temperature determined from (a).

S13: Weak Antilocalization for the field out-of-plane

The magnetoconductance at 2 K of a EuO_x/KTO(110) and an AlO_x/KTO(110) sample are shown when the field is applied out-of-plane in Fig. S13. Both samples show similar weak anti-localization features which is consistent with previous reports (*45*).

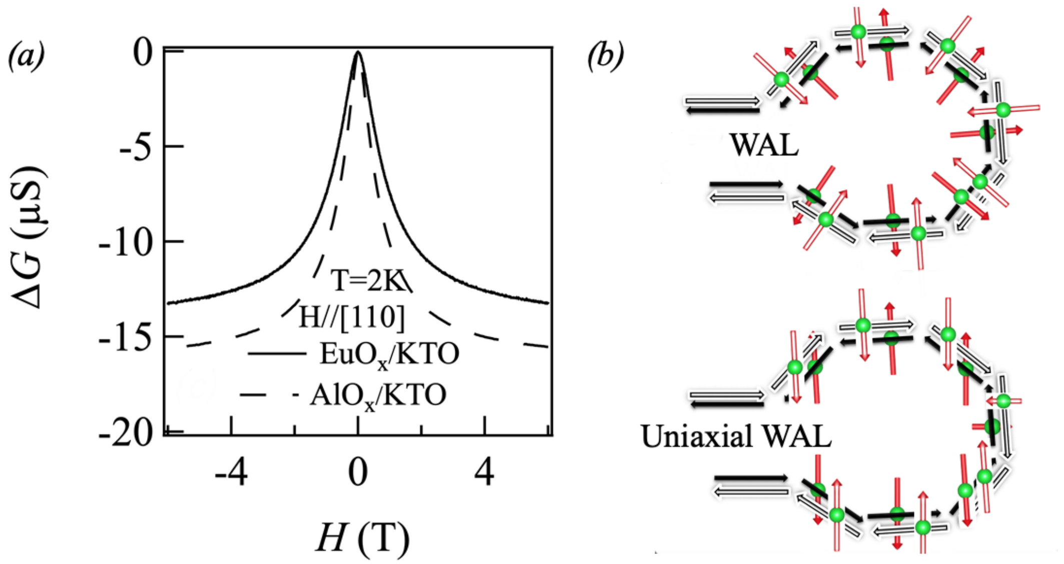

Figure S13 (a) Field dependence of the magneto-conductance at 2K for EuO_x/KTO and AlO_x/KTO. The field is applied along the [110] direction which is the *out-of-plane* direction. (b) A comparison of spin orientation due to spin-momentum locking in conventional (upper panel) and uniaxial (lower panel) weak -antilocalization, where the latter is a consequence of the 'half-Rashba' spin texture.

S14: Theoretical Considerations

A. KTO (110) electronic structure

Due to quantum confinement, the (110) surface has the special property of having low lying *xz*/*yz* states with the *xy* states pushed to higher energy. Considering the *xz*/*yz* subspace, then in the local limit in the presence of spin-orbit, one has spin and orbital moments anti-aligned with equal magnitudes due to Hund's rules, that is $L + 2S = 0$. The net result is that the total moment is quenched. This picture changes once hopping terms are introduced in the Hamiltonian. Given the large band gap in KTO, we can confine to the Ta $5d$ t_{2g} states, noting that the Ta $5d$ e_g states are much higher in energy and so can ignored in an effective low energy model. The dominant t_{2g} hopping, t, is the Ta-Ta near-neighbor one (*xz* to *xz* along the *x* and *z* bonds, etc.). For the (110) surface, one of the near-neighbor bond directions is in plane ([001]) and the other two ([100] and [010]) connect planes. This gives rise to a higher Fermi velocity along [001] than along [1-10],

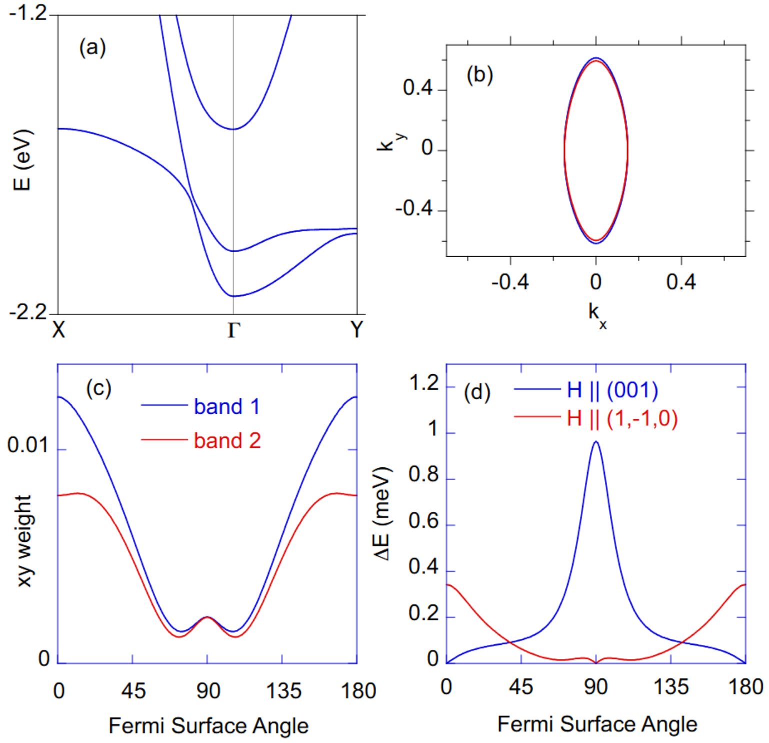

Figure S14. (a) Band dispersion for the trilayer case. X is the zone boundary along [001] and Y the zone boundary along [1-10]. The parameters are t = 0.4975 eV; t' = 0.035 eV; t'' = 0.09 eV; t''' = 0.0175 eV; and λ_{SO} = 0.265 eV. (b) Fermi surface for a density of 6 x 10^{13} cm^{-2} (chemical potential $\mu = 0.1321\ eV$). A Rashba interaction (t_R = 2 meV) has been added to the Hamiltonian to lift the Kramers degeneracy, with the Rashba-split surfaces in red and blue. The *x*-axis is along [001] and the *y*-axis along [1-10] with ±1 marking the zone boundaries. (c) Admixture of *xy* states around the Fermi surface. Bands 1 and 2 are the Rashba-split surfaces shown in (b). (d) Zeeman splitting around the Fermi surface for the two in-plane field directions. A Zeeman field of 1 meV is assumed, which for g = 2 would correspond to a 2 meV splitting. Results are shown for band 1.

resulting in the elongated Fermi surface shown in Fig. 1(b) of the main text. Including just *t* and the spin-orbit coupling, one finds that the moment texture is completely uniaxial in-plane. That is, the moments are either parallel or antiparallel to the [001] direction since [001] is the quantization axis for *xz*/*yz* moments. The resulting texture around the Fermi surface has the form $-k_y\sigma_x$ where *x* is along [001] and *y* along [1-10]. That is, it is "half" the full Rashba form of $k_x\sigma_y$ - $k_y\sigma_x$. Inclusion of other hoppings does not change this picture except when including terms that are off-diagonal in the orbital index. When including this last, a $k_x\sigma_y$ term appears but the net result is that the moment texture is strongly uniaxial, as shown in Fig. 1(b) of the main text. For simulations, we have done both the bilayer and trilayer cases including four hopping terms, *t*, *t'* (near-neighbor *xz* to *xz* along y, etc.), *t"* (next-near-neighbor hopping diagonal in the orbital index) and *t'''* (next-near-neighbor hopping off-diagonal in the orbital index). These hoppings are illustrated in the paper by Xiao *et al.* (*61*) Values were taken by fitting the bulk band structure of KTO as given in the Materials Project (*62*). Results shown were obtained by solving an 18 x 18 secular matrix (three

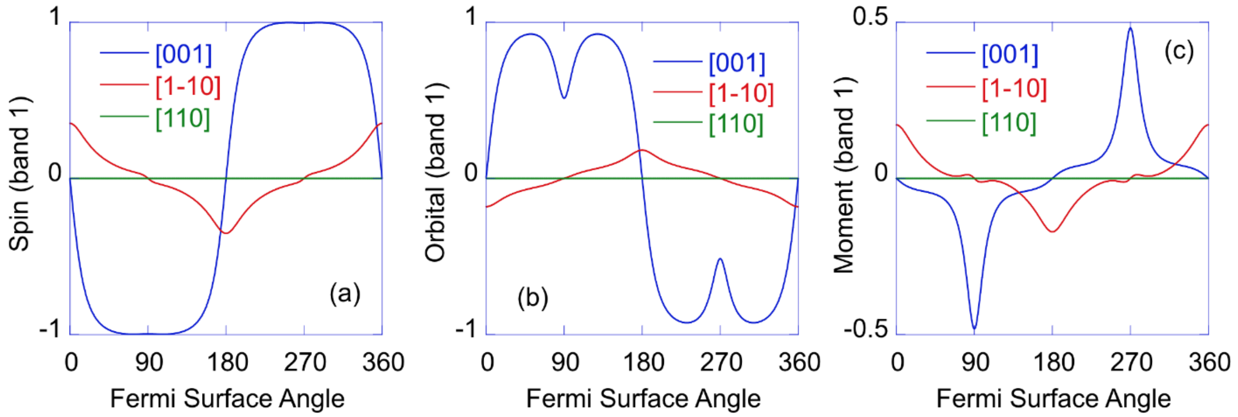

Figure S15. (a)-(c) The three components of (a) *S*, (b) *L* and (c) the moment vector (*L*+2*S*) around the band 1 Fermi surface. The maximum allowed would be ±1. Note the band 2 surface has opposite helicity.

t_{2g} orbitals per Ta site, three layers for the trilayer case, plus spin). The Zeeman term in the Hamiltonian (for a non-zero field) involves both spin and orbital contributions(*26*).

In Figure S14, we show results for the trilayer case for (a) the band dispersion, (b) the Fermi surface (corresponding to a carrier density of 6 x 10^{13} cm^{-2}), (c) the admixture of the *xy* orbitals around the Fermi surface, and (d) the Zeeman splitting around the Fermi surface assuming a Zeeman field of 1 meV. For these plots, a small Rashba interaction of 2 meV has been added to the Hamiltonian to lift the Kramers degeneracy at zero field. Note that the Zeeman splitting for fields along [1-10] follows the *xy* admixture showing its different origin from that along [001]. In Figure S15, the angular dependence of the spin, orbital, and total moment for the three directions is shown around the Fermi surface based on the results from Figure S15. Note that there is no out-of-plane contribution like there is for the (111) surface (*26*). In Table 1, we show the expectation values averaged around the Fermi surface associated with *S* and *M* for both the bilayer and trilayer cases for the two in-plane field directions, [001] and [1-10], where *M* is the total moment (2*S* + *L*, where 2 is the spin *g*-factor). This illustrates the strong anisotropy of the texture with the maximal response being for a field along [001] as expected. The anisotropy is much larger when considering only the spin texture, and the total moment texture is largely quenched due to spin/orbital moment

i	***S*** **(2L)**	***M*** **(2L)**	***S*** **(3L)**	***M*** **(3L)**
**[001]**	0.882	0.113	0.882	0.162
**[1-10]**	0.165	0.108	0.173	0.077
**[110]**	0.0	0.0	0.0	0.0

Table 1. Band 1 averages of the ith component of S and M around the Fermi surface comparing the bilayer simulation (2L) to the trilayer simulation (3L). Here $S^2 = \langle S_i^2 \rangle_{FS}$ and $M^2 = \langle M_i^2 \rangle_{FS}$ (where $M_i = L_i + 2S_i$). The maximum allowed value is 1. Note the large anisotropy for M, especially for the trilayer case, and the even larger anisotropy associated with S.

compensation given their opposing signs. When considering these results for the following two subsections, S and L for Ta can in principle couple differently to the Eu ions (that is, not necessarily as $2S + L$ with a typical exchange model involving only S).

B. In-plane upper critical field anisotropy

The basic formalism we employ is that of Fischer (*44*) based on WHH (*39*) theory.The specific formulas employed are for in-plane fields, meaning that the field for the orbital part enters quadratically. Fischer's generalization of WHH is to allow for an extra exchange field in the Zeeman term that accounts for the interaction of the Ta 5*d* electrons at the Fermi level with the Eu spins. The one generalization we do is to allow the g-factor of the Ta electrons to deviate from 2. The resulting formula for the critical field is:

$$ln\left(\frac{1}{t}\right) = \left(\frac{1}{2} + i\frac{\lambda_{so}}{4\gamma}\right)\psi\left(\frac{1}{2} + \frac{h^2 + (\frac{\lambda_{so}}{2}) + i\gamma}{2t}\right) + \left(\frac{1}{2} - i\frac{\lambda_{so}}{4\gamma}\right)\psi\left(\frac{1}{2} + \frac{h^2 + \frac{\lambda_{so}}{2} - i\gamma}{2t}\right) - \psi\left(\frac{1}{2}\right) \qquad \textbf{Eq. 1}$$

where t is the reduced temperature, T/T_c (with T_c the zero field value), λ_{so} is the spin-orbit scattering parameter ($\lambda_{so}^{-1} = 3\pi k_B T_c \tau_{so}/2\hbar$ where τ_{so} is the spin-orbit scattering time), $\psi(x)$ is the digamma function, and

$$\gamma = \sqrt{\{\alpha^2\left[\left(\frac{g}{2}\right)h + h_J\right]^2 - (\frac{\lambda_{so}}{2})^2\}} \qquad \textbf{Eq. 2}$$

where α is the Maki parameter (for thin films in a parallel field, its definition differs from the typically quoted bulk/perpendicular field value, α_B: $\alpha = 29.058\ \mathrm{sqrt}(\alpha_B/T_c)/d$ where d is the superconducting film thickness in nm, T_c is in Kelvin, and $\alpha_B = \hbar/(2m^*D)$ with m* the effective mass and D the diffusion constant), h is a reduced field, and h_J is the reduced exchange field between the polarized Eu ions and the Ta conduction electrons. For H in Tesla and T in Kelvin, $h = 0.2158\ H/(\alpha\ T_c)$. The exchange field is $h_J = h_{J0}B_J(x)$ where $B_J(x)$ is the Brillouin function for $J = 7/2$ and $x = J\frac{g'\mu_B B}{k_B T}$ with g′=2. The above formula is then optimized over the fit variables using Powell's method (*63*).

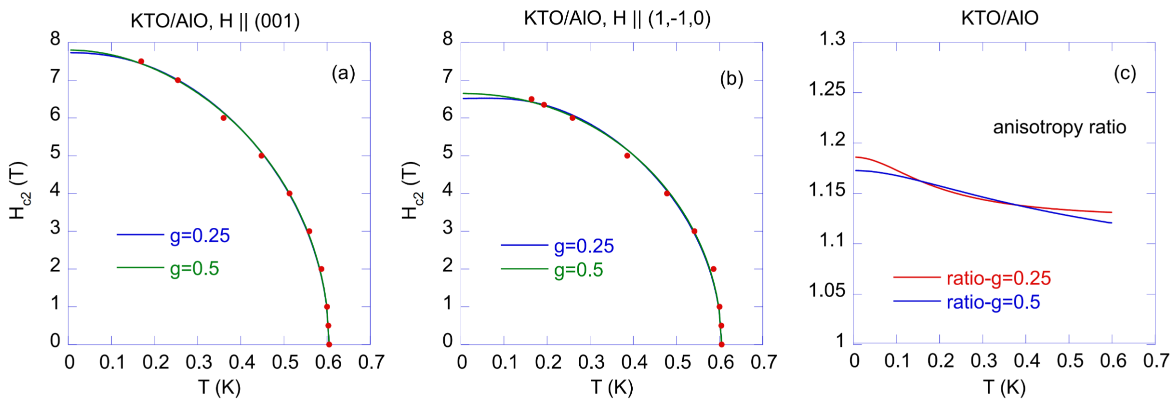

Figure S16 Fits to the upper critical field vs T for AlO_x/KTO for two different values of g for (a) fields along [001] and (b) fields along [1-10]. (c) Anisotropy ratio versus temperature for g = 0.25 and g = 0.5.

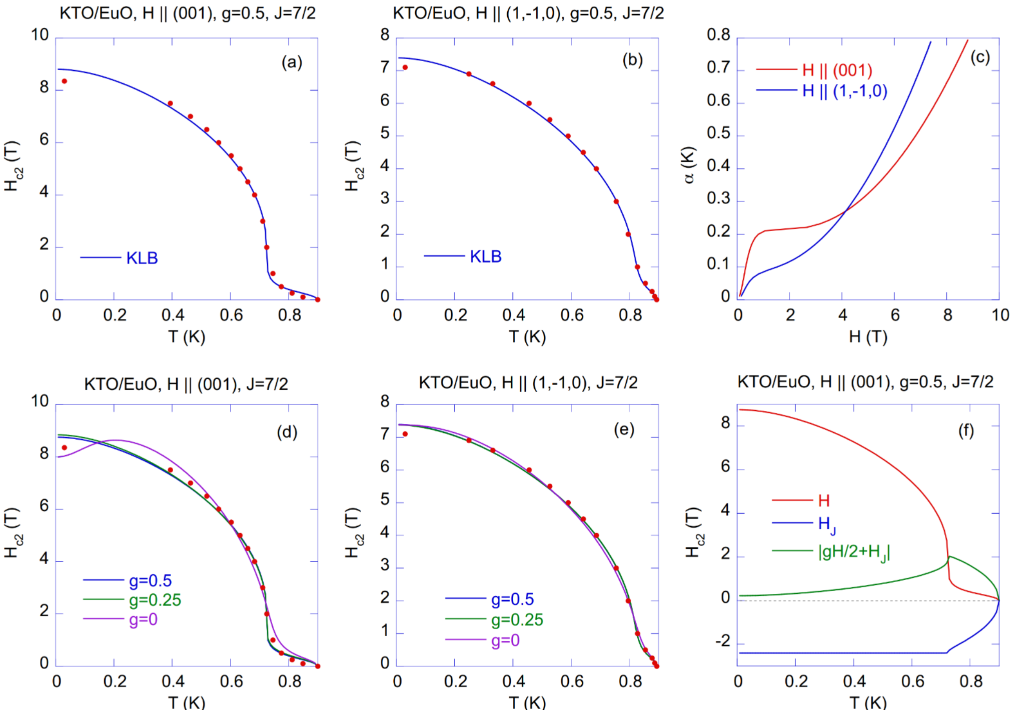

Figure S17. Fits to H_{c2} vs T for EuO/KTO (110) using the KLB formalism for (a) fields along [001] and (b) fields along [1-10]. The *g*-factor is assumed to be 0.5 and the impurity Eu^{2+} ion is assumed to have J = 7/2. (c) Plot of the pair-breaking parameter α obtained from the KLB fits. (d,e) WHH fits to the same data, for *g*-factors of 0, 0.25 and 0.5, and J = 7/2 for the Eu^{2+} ion. (f) Decomposition of H_{c2} for fields along (001) showing the exchange contribution (H_J) and the total Zeeman contribution ($gH/2+H_J$).

Figure	g	λ_{SO}	α	H_{J0}
16(a)	0.25	0.36	7.22	
16(a)	0.5	1.50	12.47	
16(b)	0.25	0.13	6.27	
16(b)	0.5	0.96	11.31	
17(d)	0.5	2.14	3.97	-2.41
17(d)	0.25	0	4.03	-1.29
17(d)	0	0	5.73	-1.07
17(e)	0.5	3.73	3.37	-1.87
17(e)	0.25	0	3.36	-0.88
17(e)	0	0	3.79	-0.72

Table 2. WHH fitting coefficients for the plots in Figs. S16 and S17. g is the g-factor, λ_{SO} the spin-orbit scattering parameter, α the Maki parameter, and H_{J0} the exchange field (Tesla).

In Figure S16, we show fits to the critical field for an AlO_x/KTO sample with T_c = 600 mK for the two in-plane field directions (where for AlO_x, h_J is not included). Given the smooth Ginzburg-Landau nature of the critical field curves, there is no unique determination of parameters (shown in Table 2) given that we have three of them (λ_{so}, α and g). For illustrative purposes, we show results for $g = 0.25$ and $g = 0.5$. Note that α differs between the two directions, which is connected to the orbital field anisotropy mentioned in the main text, with H_{c2} scaling with α. The anisotropy ratio between the two directions displays a modest temperature dependence. The results are consistent with a finite but suppressed g-factor (g acts to suppress H_{c2} at low temperatures) and an anisotropic λ_{SO}. But the anisotropy of the spin texture is difficult to infer from these measurements, unlike for our observations on EuO/KTO.

In Figure S17, we show fits to the critical field for a EuO/KTO sample with T_c = 900 mK using both KLB and WHH formalisms, where we include the exchange term h_J between Eu^{2+} ions and electrons in the KTO (110) 2DEG. For the KLB fits, we assume $g = 0.5$. For the WHH fits, we have included fits for $g = 0$, 0.25 and 0.5. In all fits, h_J is larger for fields along [001] than for [1-10], as expected. When g is non-zero, the sign of h_J matters, and we find that for the best fits, it is negative relative to h. This allows for the inflection behavior in H_c (due to rapid rise of the Brillouin function with H) to become even more pronounced due to the Jaccarino-Peter effect(*64*) . This effect is less pronounced for fields along [1-10] reflecting the moment anisotropy shown in Table 2.

C. Quantum corrections to the normal state magnetoresistance

For parallel fields, the primary contribution to weak antilocalization corrections to the magnetoresistance (MR) is the Zeeman term (*49*), noting that orbital contributions to the MR exist that will be quadratic in field. Therefore, as outlined in the main text, H_J will enter this Zeeman term as well. This was elucidated earlier for a $NdTiO_3/SrTiO_3$ 2DEG (*48*). We assume an exchange field from the Eu ion as in the previous section.

We take the quadratic orbital contribution into consideration and fit the magnetoconductance for both H//[001] and H//[1-10] using the following:

$$\frac{\Delta G}{G}(H_{\parallel}) = m_3 {H_{\parallel}}^2 + m_1 ln(1 + m_2((\frac{g}{2}) H + H_J(x))^2) \qquad \textbf{Eq 3}$$

where the first term on the right corresponds to the orbital correction and the second term to the Zeeman correction. Here, we set g=0.5 and use H_J from the g=0.5 fits shown in Fig. S17 with J=7/2. The fitted plot is shown in Figure S18 and the fitting parameters are listed in Table. 3. The fits are quite good indicating that the values found from the H_c analysis and the MR analysis are consistent with one another. Since our analysis of the anisotropy is independent of the values of τ_{SO} and τ_{φ}, we decided not to carry out the parameterization process needed to extract these parameters.

	H//[001]	H//[1-10]
m_1	-0.0031922	-0.0040034
m_2	26.189	5.1763
m_3	-0.00091977	-0.0015494

Table 3. Fitting parameters for WAL for both *H*//[001] and *H*//[1-10] from Figure S18.

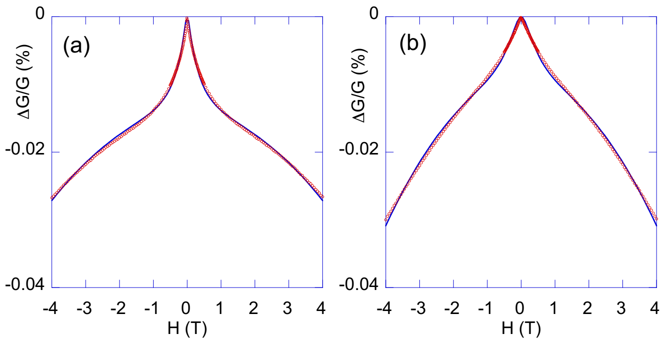

Figure S18 (a) (b) Fitting of the magnetoconductance at 2K for H//[001] and H//[1-10] respectively for EuO_x/KTO (110)

Data availability:

All data for figures in the Supplemental Materials are available via the Harvard Dataverse at the following link: https://doi.org/10.7910/DVN/VYIOWB.

References:

All literature cited in the Supplementary Materials are listed in the References section of the main manuscript.

REFERENCES

1. G. Bihlmayer, P. Noël, D. V. Vyalikh, E. V. Chulkov, A. Manchon, Rashba-like physics in condensed matter. *Nat. Rev. Phys.* **4**, 642–659 (2022).

2. S. Picozzi, Spin–orbit coupling in quantum materials: Emergent phenomena, their modelling and examples from two-dimensional magnets. *Riv. Nuovo Cimento.* **47**, 609–652 (2024).

3. Y. A. Bychkov, E. I. Rashba, Properties of a 2D electron-gas with lifted spectral degeneracy. *JETP Lett.* **39**, 78–81 (1984).

4. M. Smidman, M. B. Salamon, H. Q. Yuan, D. F. Agterberg, Superconductivity and spin–orbit coupling in non-centrosymmetric materials: A review. *Rep. Prog. Phys.* **80**, 036501 (2017).

5. L. P. Gor'kov, E. I. Rashba, Superconducting 2D system with lifted spin degeneracy: Mixed singlet-triplet state. *Phys. Rev. Lett.* **87**, 037004 (2001).

6. J. M. Lu, O. Zheliuk, I. Leermakers, N. F. Q. Yuan, U. Zeitler, K. T. Law, J. T. Ye, Evidence for two-dimensional Ising superconductivity in gated MoS_2. *Science* **350**, 1353–1357 (2015).

7. Y. Saito, Y. Nakamura, M. S. Bahramy, Y. Kohama, J. Ye, Y. Kasahara, Y. Nakagawa, M. Onga, M. Tokunaga, T. Nojima, Y. Yanase, Y. Iwasa, Superconductivity protected by spin–valley locking in ion-gated MoS_2. *Nat. Phys.* **12**, 144–149 (2016).

8. J. Falson, Y. Xu, M. Liao, Y. Zang, K. Zhu, C. Wang, Z. Zhang, H. Liu, W. Duan, K. He, H. Liu, J. H. Smet, D. Zhang, Q.-K. Xue, Type-II Ising pairing in few-layer stanene. *Science* **367**, 1454–1457 (2020).

9. B. T. Zhou, N. F. Q. Yuan, H. L. Jiang, K. T. Law, Ising superconductivity and Majorana fermions in transition-metal dichalcogenides. *Phys. Rev. B* **93**, 180501 (2016).

10. K. Matano, M. Kriener, K. Segawa, Y. Ando, G. Q. Zheng, Spin-rotation symmetry breaking in the superconducting state of $Cu_xBi_2Se_3$. *Nat. Phys.* **12**, 852–854 (2016).

11. A. Hamill, B. Heischmidt, E. Sohn, D. Shaffer, K. T. Tsai, X. Zhang, X. X. Xi, A. Suslov, H. Berger, L. Forró, F. J. Burnell, J. Shan, K. F. Mak, R. M. Fernandes, K. Wang, V. S. Pribiag, Two-fold symmetric superconductivity in few-layer $NbSe_2$. *Nat. Phys.* **17**, 949–954 (2021).

12. A. Johansson, J. Henk, I. Mertig, Theoretical aspects of the Edelstein effect for anisotropic two-dimensional electron gas and topological insulators. *Phys. Rev. B* **93**, 195440 (2016).

13. S. M. Frolov, M. J. Manfra, J. D. Sau, Topological superconductivity in hybrid devices. *Nat. Phys.* **16**, 718–724 (2020).

14. M. M. Desjardins, L. C. Contamin, M. R. Delbecq, M. C. Dartiailh, L. E. Bruhat, T. Cubaynes, J. J. Viennot, F. Mallet, S. Rohart, A. Thiaville, A. Cottet, T. Kontos, Synthetic spin-orbit interaction for Majorana devices. *Nat. Mater.* **18**, 1060–1064 (2019).

15. J. Linder, J. W. A. Robinson, Superconducting spintronics. *Nat. Phys.* **11**, 307–315 (2015).

16. M. Amundsen, J. Linder, J. W. A. Robinson, I. Zutic, N. Banerjee, Spin-orbit effects in superconducting hybrid structures. *Rev. Mod. Phys.* **96**, 021003 (2024).

17. C. Liu, X. Yan, D. Jin, Y. Ma, H.-W. Hsiao, Y. Lin, T. M. Bretz-Sullivan, X. Zhou, J. Pearson, B. Fisher, J. S. Jiang, W. Han, J.-M. Zuo, J. Wen, D. D. Fong, J. Sun, H. Zhou, A. Bhattacharya, Two-dimensional superconductivity and anisotropic transport at $KTaO_3$ (111) interfaces. *Science* **371**, 716–721 (2021).

18. Z. Chen, Z. R. Liu, Y. Q. Sun, X. X. Chen, Y. Liu, H. Zhang, H. K. Li, M. Zhang, S. Y. Hong, T. S. Ren, C. Zhang, H. Tian, Y. Zhou, J. R. Sun, Y. W. Xie, Two-dimensional superconductivity at the $LaAlO_3/KTaO_3$ (110) heterointerface. *Phys. Rev. Lett.* **126**, 026802 (2021).

19. C. Liu, X. Zhou, D. Hong, B. Fisher, H. Zheng, J. Pearson, J. S. Jiang, D. Jin, M. R. Norman, A. Bhattacharya, Tunable superconductivity and its origin at $KTaO_3$ interfaces. *Nat. Commun.* **14**, 951 (2023).

20. E. A. Martínez, J. Dai, M. Tallarida, N. M. Nemes, F. Y. Bruno, Anisotropic electronic structure of the 2D electron gas at the $AlO_x/KTaO_3$(110) interface. *Adv. Electron. Mater.* **9**, 2300267 (2023).

21. P. W. Krantz, A. Tyner, P. Goswami, V. Chandrasekhar, Nonlinear hall effect in $KTaO_3$ two-dimensional electron gases. arXiv:2411.09161 (2024).

22. S. J. Poage, X. S. Gao, M. Baksi, S. Salmani-Rezaie, D. A. Muller, D. P. Kumah, C. N. Lau, J. Lorenzana, M. N. Gastiasoro, K. Ahadi, Violation of the Pauli limit at KTaO (110) interfaces. *Phys. Rev. B* **111**, 214506 (2025).

23. F. Y. Bruno, S. M. Walker, S. Riccò, A. de la Torre, Z. M. Wang, A. Tamai, T. K. Kim, M. Hoesch, M. S. Bahramy, F. Baumberger, Band structure and spin-orbital texture of the (111)-$KTaO_3$ 2D electron gas. *Adv. Electron Mater.* **5**, 1800860 (2019).

24. S. Varotto, A. Johansson, B. Göbel, L. M. Vicente-Arche, S. Mallik, J. Bréhin, R. Salazar, F. Bertran, P. Le Fèvre, N. Bergeal, J. Rault, I. Mertig, M. Bibes, Direct visualization of Rashba-split bands and spin/orbital-charge interconversion at $KTaO_3$ interfaces. *Nat. Commun.* **13**, 6165 (2022).

25. S. Sugano, Y. Tanabe, H. Kamimura, *Multiplets of Transition-Metal Ions in Crystals* (Elsevier Science, 1970).

26. A. H. Al-Tawhid, S. J. Poage, S. Salmani-Rezaie, A. Gonzalez, S. Chikara, D. A. Muller, D. Kumah, M. N. Gastiasoro, J. Lorenzana, K. Ahadi, Enhanced critical field of superconductivity at an oxide interface. *Nano Lett.* **23**, 6944–6950 (2023).

27. G. Singh, A. Jouan, G. Herranz, M. Scigaj, F. Sánchez, L. Benfatto, S. Caprara, M. Grilli, G. Saiz, F. Couëdo, C. Feuillet-Palma, J. Lesueur, N. Bergeal, Gap suppression at a Lifshitz transition in a multi-condensate superconductor. *Nat. Mater.* **18**, 948–954 (2019).

28. A. Annadi, Q. Zhang, X. Renshaw Wang, N. Tuzla, K. Gopinadhan, W. M. Lü, A. Roy Barman, Z. Q. Liu, A. Srivastava, S. Saha, Y. L. Zhao, S. W. Zeng, S. Dhar, E. Olsson, B. Gu,

S. Yunoki, S. Maekawa, H. Hilgenkamp, T. Venkatesan, Ariando, Anisotropic two-dimensional electron gas at the $LaAlO_3/SrTiO_3$ (110) interface. *Nat.Commun.* **4**, 1838 (2013).

29. Z. Wang, Z. Zhong, X. Hao, S. Gerhold, B. Stöger, M. Schmid, J. Sánchez-Barriga, A. Varykhalov, C. Franchini, K. Held, U. Diebold, Anisotropic two-dimensional electron gas at $SrTiO_3$ (110). *Proc. Natl. Acad. Sci. U.S.A.* **111**, 3933–3937 (2014).

30. P. Moetakef, J. R. Williams, D. G. Ouellette, A. P. Kajdos, D. Goldhaber-Gordon, S. J. Allen, S. Stemmer, Carrier-controlled ferromagnetism in $SrTiO_3$. *Phys. Rev. X* **2**, 021014 (2012).

31. D. A. Dikin, M. Mehta, C. W. Bark, C. M. Folkman, C. B. Eom, V. Chandrasekhar, Coexistence of superconductivity and ferromagnetism in two dimensions. *Phys. Rev. Lett.* **107**, 056802 (2011).

32. F. Wen, Y. Cao, X. Liu, B. Pal, S. Middey, M. Kareev, J. Chakhalian, Evolution of ferromagnetism in two-dimensional electron gas of $LaTiO_3/SrTiO_3$. *Appl. Phys. Lett.* **112**, 122405 (2018).

33. P. W. Krantz, A. Tyner, P. Goswami, V. Chandrasekhar, Intrinsic magnetism in $KTaO_3$ heterostructures. *Appl. Phys. Lett.* **124**, 093102 (2024).

34. X. Hua, Z. Zeng, F. Meng, H. Yao, Z. Huang, X. Long, Z. Li, Y. Wang, Z. Wang, T. Wu, Z. Weng, Y. Wang, Z. Liu, Z. Xiang, X. Chen, Superconducting stripes induced by ferromagnetic proximity in an oxide heterostructure. *Nat. Phys.* **20**, 957–963 (2024).

35. K. Maki, T. Tsuneto, Pauli paramagnetism and superconducting state. *Prog. Theor. Phys.* **31**, 945–956 (1964).

36. Z. Zhang, W. Jiang, T. Shao, Y. Qiao, X. Chen, Q. Zhao, M. Chen, R. Dou, C. Xiong, J. Nie, A spin–orbit scattering–enhanced high upper critical field at the $LaAlO_3/KTaO_3$(111) superconducting interface. *New J. Phys.* **25**, 023023 (2023).

37. E. G. Arnault, A. H. Al-Tawhid, S. Salmani-Rezaie, D. A. Muller, D. P. Kumah, M. S. Bahramy, G. Finkelstein, K. Ahadi, Anisotropic superconductivity at $KTaO_3$ (111) interfaces. *Sci. Adv.* **9**, eadf1414 (2023).

38. M. Tinkham, *Introduction to Superconductivity* (Dover Publications, 2004).

39. N. R. Werthamer, E. Helfand, P. C. Hohenberg, Temperature and purity dependence of the superconducting critical field, H_{c2}. III. Electron spin and spin-orbit effects. *Phys. Rev.* **147**, 295–302 (1966).

40. O. Fischer, M. Decroux, S. Roth, R. Chevrel, M. Sergent, Compensation of the paramagnetic effect on H_{c2} by magnetic moments: 700 kG superconductors. *J. Phys. C. Solid Stat. Phys.* **8**, L474–L477 (1975).

41. H. Xu, H. Li, N. Gauquelin, X. Chen, W.-F. Wu, Y. Zhao, L. Si, D. Tian, L. Li, Y. Gan, S. Qi, M. Li, F. Hu, J. Sun, D. Jannis, P. Yu, G. Chen, Z. Zhong, M. Radovic, J. Verbeeck, Y. Chen, B. Shen, Giant tunability of rashba splitting at cation-exchanged polar oxide interfaces by selective orbital hybridization. *Adv. Mater.* **36**, e2313297 (2024).

42. B. Lama, E. Y. Tsymbal, T. R. Paudel, Effects of intermixing and oxygen vacancies on a two-dimensional electron gas at the polar $TbScO_3/KTaO_3$ (001) interface. *Phys. Rev. Mater.* **7**, 026201 (2023).

43. R. A. Klemm, A. Luther, M. R. Beasley, Theory of upper critical-field in layered superconductors. *Phys. Rev. B* **12**, 877–891 (1975).

44. O. H. Fischer, Properties of high-field superconductors containing localized magnetic moments. *Helv. Phys. Acta* **45**, 331–397 (1972).

45. Y. Gan, F. Yang, L. Kong, X. Chen, H. Xu, J. Zhao, G. Li, Y. Zhao, L. Yan, Z. Zhong, Y. Chen, H. Ding, Light-induced giant rashba spin–orbit coupling at superconducting $KTaO_3$ (110) heterointerfaces. *Adv. Mater.* **35**, e2300582 (2023).

46. A. H. Al-Tawhid, J. Kanter, M. Hatefipour, D. P. Kumah, J. Shabani, K. Ahadi, Superconductivity and weak anti-localization at $KTaO_3$ (111) interfaces. *J. Electron. Mater.* **51**, 6305–6309 (2022).

47. X. Hua, F. Meng, Z. Huang, Z. Li, S. Wang, B. Ge, Z. Xiang, X. Chen, Tunable two-dimensional superconductivity and spin-orbit coupling at the EuO/$KTaO_3$(110) interface. *NPJ Quant. Mater.* **7**, 97 (2022).

48. X. Cai, Y. Ayino, J. Yue, P. Xu, B. Jalan, V. S. Pribiag, Disentangling spin-orbit coupling and local magnetism in a quasi-two-dimensional electron system. *Phys. Rev. B* **100**, 081402 (2019).

49. S. Maekawa, H. Fukuyama, Magnetoresistance in two-dimensional disordered systems: Effects of zeeman splitting and spin-orbit scattering. *J. Physical Soc. Japan* **50**, 2516–2524 (1981).

50. F. F. Balakirev, T. Kong, M. Jaime, R. D. McDonald, C. H. Mielke, A. Gurevich, P. C. Canfield, S. L. Bud'ko, Anisotropy reversal of the upper critical field at low temperatures and spin-locked superconductivity in $K_2Cr_3As_3$. *Phys. Rev. B* **91**, 220505 (2015).

51. J. K. Bao, J. Y. Liu, C. W. Ma, Z. H. Meng, Z. T. Tang, Y. L. Sun, H. F. Zhai, H. Jiang, H. Bai, C. M. Feng, Z. A. Xu, G. H. Cao, Superconductivity in quasi-one-dimensional $K_2Cr_3As_3$ with significant electron correlations. *Phys. Rev. X* **5**, 011013 (2015).

52. H. K. Zuo, J. K. Bao, Y. Liu, J. H. Wang, Z. Jin, Z. C. Xia, L. Li, Z. Xu, J. Kang, Z. W. Zhu, G. H. Cao, Temperature and angular dependence of the upper critical field in $K_2Cr_3As_3$. *Phys. Rev. B* **95**, 014502 (2017).

53. J. Yang, J. Luo, C. Yi, Y. Shi, Y. Zhou, G. Q. Zheng, Spin-triplet superconductivity in $K_2Cr_3As_3$. *Sci. Adv.* **7**, eabl4432 (2021).

54. I. Silber, S. Mathimalar, I. Mangel, A. K. Nayak, O. Green, N. Avraham, H. Beidenkopf, I. Feldman, A. Kanigel, A. Klein, M. Goldstein, A. Banerjee, E. Sela, Y. Dagan, Two-component nematic superconductivity in 4Hb-TaS_2. *Nat. Commun.* **15**, 824 (2024).

55. Y. M. Xie, B. T. Zhou, K. T. Law, Spin-orbit-parity-coupled superconductivity in topological monolayer WTe_2. *Phys. Rev. Lett.* **125**, 107001 (2020).

56. J. Cui, P. Li, J. Zhou, W. Y. He, X. Huang, J. Yi, J. Fan, Z. Ji, X. Jing, F. Qu, Z. G. Cheng, C. Yang, L. Lu, K. Suenaga, J. Liu, K. T. Law, J. Lin, Z. Liu, G. Liu, Transport evidence of

asymmetric spin-orbit coupling in few-layer superconducting $1T_d$-$MoTe_2$. *Nat. Commun.* **10**, 2044 (2019).

57. E. Z. Zhang, Y. M. Xie, Y. Q. Fang, J. L. Zhang, X. Xu, Y. C. Zou, P. L. Leng, X. J. Gao, Y. Zhang, L. F. Ai, Y. D. Zhang, Z. H. Jia, S. S. Liu, J. Y. Yan, W. Zhao, S. J. Haigh, X. F. Kou, J. S. Yang, F. Q. Huang, K. T. Law, F. X. Xiu, S. M. Dong, Spin-orbit-parity coupled superconductivity in atomically thin 2M-WS_2. *Nat. Phys.* **19**, 106–113 (2023).

58. P. He, S. M. Walker, S. S. L. Zhang, F. Y. Bruno, M. S. Bahramy, J. M. Lee, R. Ramaswamy, K. Cai, O. Heinonen, G. Vignale, F. Baumberger, H. Yang, Observation of out-of-plane spin texture in a $SrTiO_3$ (111) two-dimensional electron gas. *Phys. Rev. Lett.* **120**, 266802 (2018).

59. H. Zhang, Y. Yun, X. Zhang, H. Zhang, Y. Ma, X. Yan, F. Wang, G. Li, R. Li, T. Khan, Y. Chen, W. Liu, F. Hu, B. Liu, B. Shen, W. Han, J. Sun, High-mobility spin-polarized two-dimensional electron gases at EuO/$KTaO_3$ interfaces. *Phys. Rev. Lett.* **121**, 116803 (2018).

60. T. Yamasaki, K. Ueno, A. Tsukazaki, T. Fukumura, M. Kawasaki, Observation of anomalous Hall effect in EuO epitaxial thin films grown by a pulse laser deposition. *Appl. Phys. Lett.* **98**, 082116 (2011).

61. D. Xiao, W. Zhu, Y. Ran, N. Nagaosa, S. Okamoto, Interface engineering of quantum Hall effects in digital transition metal oxide heterostructures. *Nat. Commun.* **2**, 596 (2011).

62. A. Jain, S. P. Ong, G. Hautier, W. Chen, W. D. Richards, S. Dacek, S. Cholia, D. Gunter, D. Skinner, G. Ceder, K. A. Persson, Commentary: The materials project: A materials genome approach to accelerating materials innovation. *APL Materials* **1**, 011002 (2013).

63. W. T. Vetterling, S. A. Teukolsky, W. H. Press, B. P. Flannery, *Numerical Recipes* (Cambridge Univ. Press, ed. 2, 1992).

64. V. Jaccarino, M. Peter, Ultra-high-field superconductivity. *Phys. Rev. Lett.* **9**, 290–292 (1962).
